# Modifications of 3D-Printed Adsorbent Materials for Pollutant Removal from Wastewaters

**DOI:** 10.3390/molecules31203738

**Published:** 2026-10-18

**Authors:** Konstantinos N. Maroulas, George Z. Kyzas

**Affiliations:** Hephaestus Laboratory, School of Chemistry, Faculty of Sciences, Democritus University of Thrace, GR-65404 Kavala, Greece; kmaroula@chem.duth.gr

**Keywords:** 3D-printing, additive manufacturing, wastewater, adsorption, modifications

## Abstract

Adsorption remains one of the most practical and widely adopted strategies for removing pollutants from wastewater, yet conventional powder and granular adsorbents suffer from persistent limitations: poor recyclability, difficult solid–liquid separation, and limited control over selectivity in complex, multi-contaminant streams. Three-dimensional (3D) printing has emerged as a compelling alternative, offering precise control over geometry, porosity, and macrostructure that conventional shaping methods simply cannot achieve. However, the base polymers and inks most commonly used in additive manufacturing, such as PLA, ABS, PETG, and alginate, are chemically inert and lack inherent affinity for specific pollutants. Achieving meaningful adsorption capacity therefore depends critically on how and when the active functional component is introduced into the printed structure. This review examines the full spectrum of modification strategies applied to 3D-printed adsorbents for wastewater treatment, organized by the timing of modification relative to printing. Pre-printing approaches, including composite filament fabrication, functional ink formulation, and resin doping with active materials such as MOFs, biochar, zeolites, and metal oxides, are critically evaluated alongside post-printing strategies, including surface coating, nanoparticle decoration, chemical grafting, plasma treatment, and in situ growth of porous frameworks. For each approach, the implications for pollutant selectivity are assessed across the main contaminant classes: heavy metals, synthetic dyes, and organic micropollutants such as pharmaceuticals and Bisphenol A, which are considered according to their charge, hydrophobicity, and adsorption mechanism. Finally, key structure–modification relationships are identified, gaps in regeneration, competitive-adsorption, and real-wastewater testing are highlighted, and directions for rational, pollutant-targeted adsorbent design using additive manufacturing are outlined.

## 1. Introduction

Water is vital for life and crucial for worldwide economic progress, since it supports several sectors such as agriculture, manufacturing, and household functions. The increasing dangers of water contamination, propelled by fast industrial growth, urban expansion, and farming methods, pose a considerable ecological threat, requiring improved protection and remediation strategies for sustainable water resource governance [1]. The pollutants present in aqueous media can vary, ranging from pesticides to heavy metals, pharmaceuticals, and organic dyes resulting from human actions. These contaminants disturb aquatic ecosystems and result in eutrophication, impacting food chains and water quality [2]. They can pose significant health hazards to both humans and animals, leading to conditions including cancer and endocrine disruption, especially in low-income countries [3]. In particular, organic contaminants frequently infiltrate the food chain via tainted seafood, whereas inorganic pollutants such as heavy metals amass in groundwater, resulting in significant health complications, particularly among children [4]. Thus, this situation has driven the scientific community to develop efficient, practical and sustainable methods for pollutant remediation.

Wastewater treatment techniques may be classified into three primary categories: physical, chemical, and biological [5]. To begin with, advanced oxidation processes (AOPs) decompose organic and inorganic pollutants, with ozonation being a prevalent method, while membrane separation utilizes filters that eliminate contaminants based on pore dimensions [6]. Electrochemical and photo-electrochemical oxidation methods and biological remediation also decompose pollutants. However, these approaches are costly, time-consuming or require a significant amount of chemicals [7,8]. On the contrary, adsorption is distinguished as a simple, economical, and versatile method that effectively addresses various contaminants, positioning it as one of the most viable alternatives in contemporary wastewater treatment technologies [9].

Traditional adsorbents each have their strengths and trade-offs. For example, activated carbon has a large surface area and works well on organic compounds and heavy metals. However, it is costly to produce, and its performance reduces after regeneration [10]. Another example is zeolites, which are a good alternative. They remove cations through selective ion exchange, and are cheaper to make than activated carbon. Still, their performance depends heavily on the zeolite type and the pollutant involved [10]. Lastly, biochar, made from agricultural waste, is cheap and sustainable, but has a lower adsorption capacity than activated carbon [11].

To alleviate these drawbacks, advanced nanomaterials have been developed. Carbon nanotubes and graphene offer a high surface area and strong interactions (π-π stacking, electrostatic attraction), making them effective for dyes and heavy metals [12]. Also, metal oxides and MOFs achieve selective adsorption through coordination bonding and molecular sieving, while magnetic nanoparticles are easy to separate from water after use [13,14]. Even with these advances, adsorption still faces important limitations. Capacity is often too low for high pollutant concentrations, or selectivity is poor, since pollutants compete for adsorption sites. In addition, materials can degrade under harsh wastewater conditions and fouling makes recovery and reuse difficult over time [15].

3D printing, or additive manufacturing, revolutionizes the synthesis of adsorbents by adding layers of material according to CAD models [16]. This rapidly evolving technology is utilized in multiple other sectors such as healthcare, agriculture, and food packaging, enabling the mass customization of intricate structures [17]. In wastewater treatment specifically, 3D printing enhances the design of customized membranes and adsorbents for efficient pollutant removal and oil/water separation [18]. The most important aspect is that it allows for tailored structures that improve pollutant removal. Also, it can provide 3D photocatalysts as alternatives to traditional materials, effectively converting hazardous contaminants. Thus, 3D printing proves to be both efficient and cost-effective in addressing environmental challenges.

**Figure 1:**
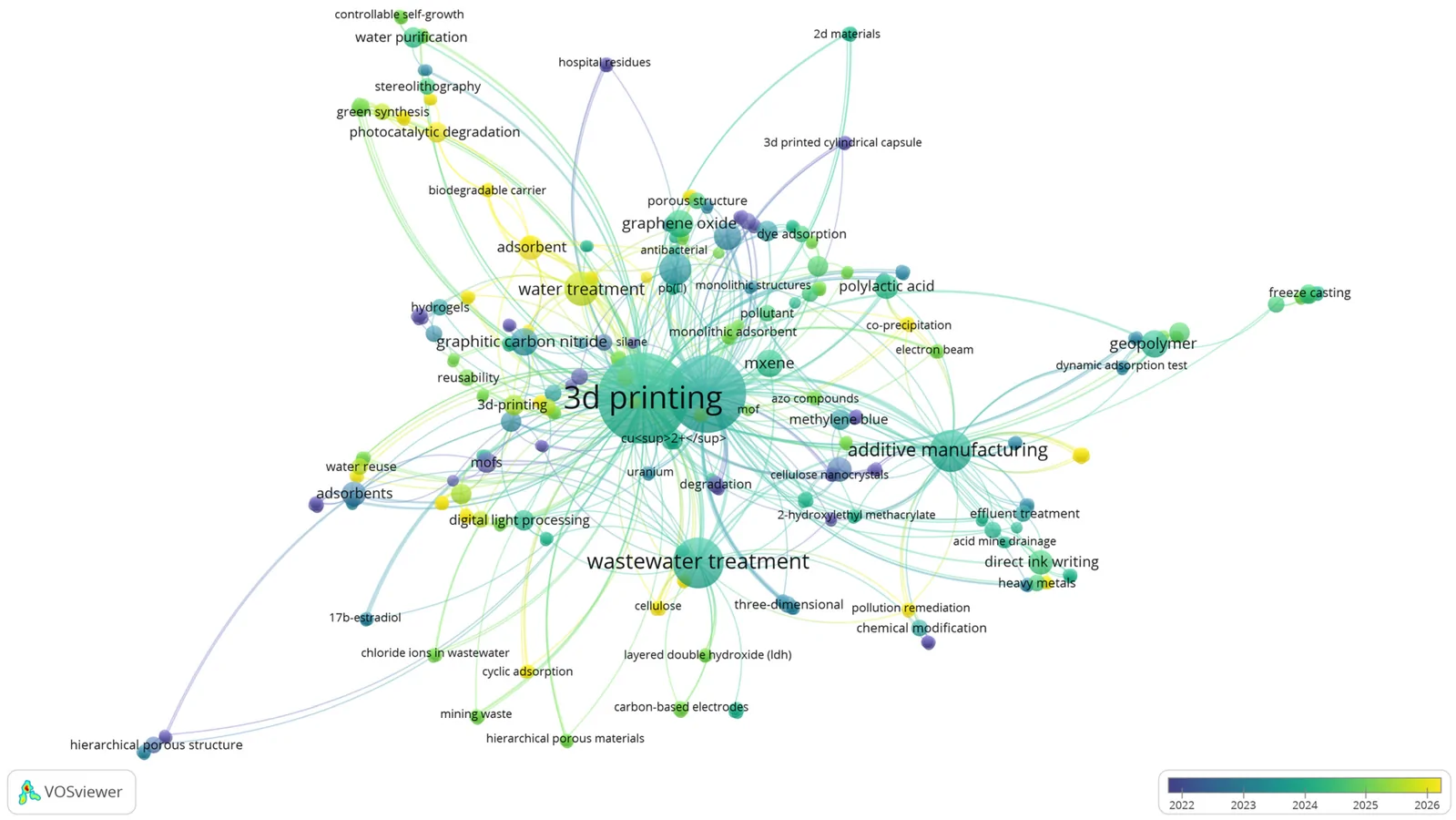
Overlay visualization of keyword co-occurrence for 3D-printed adsorbents in wastewater treatment, generated with VOSviewer (Scopus, 136 documents, 2013–2026). Node size reflects keyword occurrence, links show co-occurrence, and color indicates the average publication year.

To map the research landscape, a keyword co-occurrence analysis was carried out in VOSviewer (version 1.6.20) [19] using documents retrieved from Scopus with the terms “3D printing”, “wastewater” and “adsorption” (136 documents, search date [2 October 2026]). The overlay map (Figure 1) is colored by the average publication year of each keyword. As expected, the network is centered on 3D printing, adsorption and wastewater treatment, which are closely linked to additive manufacturing. Around this core, the most frequent active phases are carbon-based and two-dimensional materials, such as graphene oxide, MXene and graphitic carbon nitride, together with MOFs, hydrogels and cellulose-based materials. Direct ink writing, digital light processing and stereolithography are the main printing routes. In addition, the target pollutants are dominated by heavy metals (Pb, Cu, U) and dyes, mainly methylene blue and azo compounds. In contrast, emerging contaminants such as hormones and hospital effluents appear only as small, weakly connected nodes, and pharmaceuticals and PFAS are almost absent. The color scale also shows a clear shift in focus. Earlier studies (2022–2023) dealt mostly with MOFs, hydrogels and dye adsorption. More recent work (2025–2026) moves towards sustainable materials, such as cellulose, biodegradable carriers and mining waste, multifunctional systems that combine adsorption with photocatalytic degradation, and practical aspects such as cyclic use and water reuse. Notably, “chemical modification” lies at the edge of the network, with few links. This suggests that modification strategies are still reported case by case rather than studied systematically. This gap is the main motivation of this review.

While 3D printing offers unprecedented control over the geometry and porosity of adsorbent structures, the printed material itself is rarely optimal for pollutant uptake straight off the printer. Most common printing feedstocks (thermoplastics, resins, ceramic pastes) were not originally developed with adsorption or catalytic performance in mind, and their surfaces often lack the functional groups, roughness, or reactive sites needed for efficient contaminant removal. This has driven growing interest in combining 3D printing with surface modification strategies, either by incorporating functional materials into the feedstock before printing (pre-printing modification) or by treating the printed structure afterwards through physical or chemical means (post-printing modification). Each approach carries its own trade-offs in terms of process complexity, scalability, and the extent to which the final structure’s mechanical integrity and printability are preserved. Several reviews have already addressed 3D printing for water treatment. Most of them are organized around printing techniques, feedstock materials, and device types (membranes, photocatalysts, and structured adsorbents), or around specific pollutant classes such as heavy metals and dyes. Surface functionalization of printed parts has also been reviewed, but either for general applications rather than water treatment, or for post-printing functionalization only, across a broad range of water-related devices such as meshes, membranes, and biocarriers. In contrast, this review focuses specifically on adsorbents and is organized according to when the functional component is introduced, i.e., before or after printing. This allows the two routes to be compared directly in terms of active-site accessibility, adsorption capacity, printability, mechanical integrity, and reusability, including the possibility of re-functionalizing the printed scaffold. In addition, rather than only summarizing reported performance, the evidence is critically appraised: adsorption is distinguished from photocatalytic degradation, demonstrated selectivity from simple removal efficiency, and molecular mechanisms from kinetic- and isotherm-model fits, while the links between printing parameters and adsorption kinetics and the techno-economic aspects of each route are also discussed. This review therefore aims to bring together the current state of the art on pre- and post-printing modification strategies for 3D-printed materials, with a particular focus on their application in wastewater treatment, and to critically examine how these strategies influence pollutant removal performance, material stability, and practical feasibility for real-world water treatment applications.

## 2. Overview of 3D Printing Techniques

3D printing processes are classified into seven categories: (1) binder jetting, such as color jetting and ExOne; (2) directed energy deposition, such as laser engineered net shaping; (3) material extrusion, such as FDM; (4) material jetting, such as polyjet and multi-jet printing; (5) powder bed fusion, such as selective laser sintering; (6) sheet lamination, such as laminated object manufacturing; and (7) vat photopolymerization, such as digital light processing, stereolithography, and two-photon polymerization (2PP) [20] in accordance with the International Organization for Standardization (ISO)/American Society for Testing and Materials (ASTM) 52900:2021 standard [21]. Table 1 summarizes key information about each 3D printing technique.

### 2.1. Vat Photopolymerization

**Figure 2:**
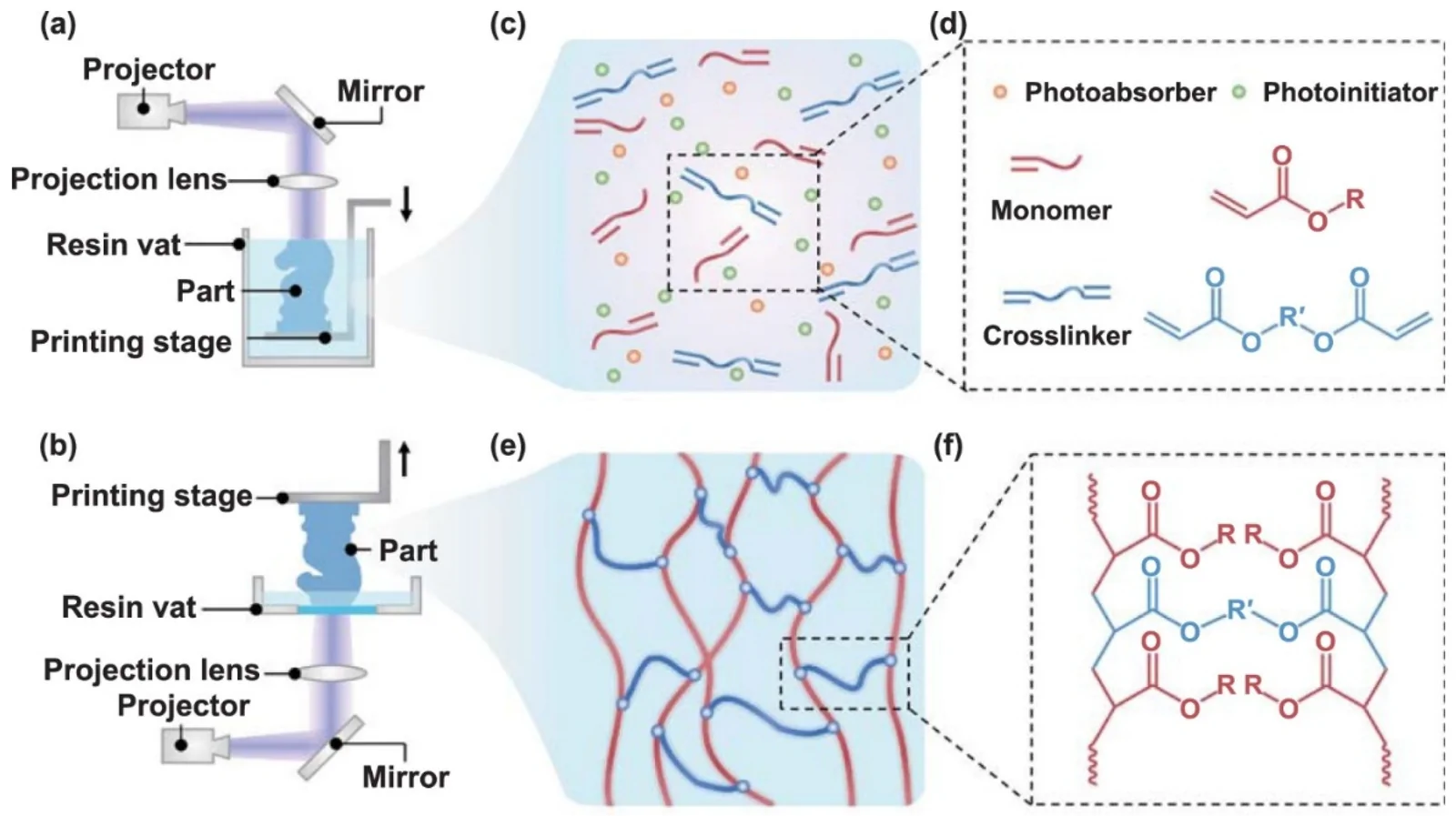
Single-material DLP 3D printing systems. (**a**) Top-down DLP 3D printing. (**b**) Bottom-up DLP 3D printing. (**c**) Schematic diagram of chemicals in liquid polymer resin. (**d**) Chemical composition of photocurable resin: monomer, crosslinker, photoinitiator and photoabsorber. (**e**) Schematic diagram of photopolymerized building blocks. (**f**) Chemical structures of crosslinked polymer networks. Reproduced with permission from [28].

The several vat photopolymerization techniques used in 3D printing use light sources to start the polymerization of photosensitive materials [18]. Digital Light Processing (DLP), Stereolithography (SLA) and Continuous Liquid Interface Production (CLIP) are important methods that are frequently employed [27]. DLP achieves faster printing speeds and a resolution of around 1 μm by using a digital projector to cure resin layer by layer. Printing resolution may be improved by including light absorbers [28]. SLA, on the other hand, produces a lesser resolution of about 10 μm but uses a UV laser for layer-wise curing [29]. Because of its special use of oxygen as a polymerization inhibitor, CLIP has become a cutting-edge technique that allows continuous printing at rates 100 times faster than DLP and SLA. However, there are numerous polymerization factors that affect the quality of printed materials, including UV intensity, scanning speed, and exposure duration [30]. For instance, when creating complex ceramic spacers for membrane filtration applications, DLP technology efficiently lowers energy consumption and increases flow. However, compared to extrusion-based techniques, SLA and DLP have limited multimaterial printing capabilities, and SLA has difficulties, particularly with regard to the mechanical robustness of printed components [31,32]. Figure 2 summarizes key DLP 3D printing systems.

### 2.2. Extrusion-Based Printing

**Figure 3:**
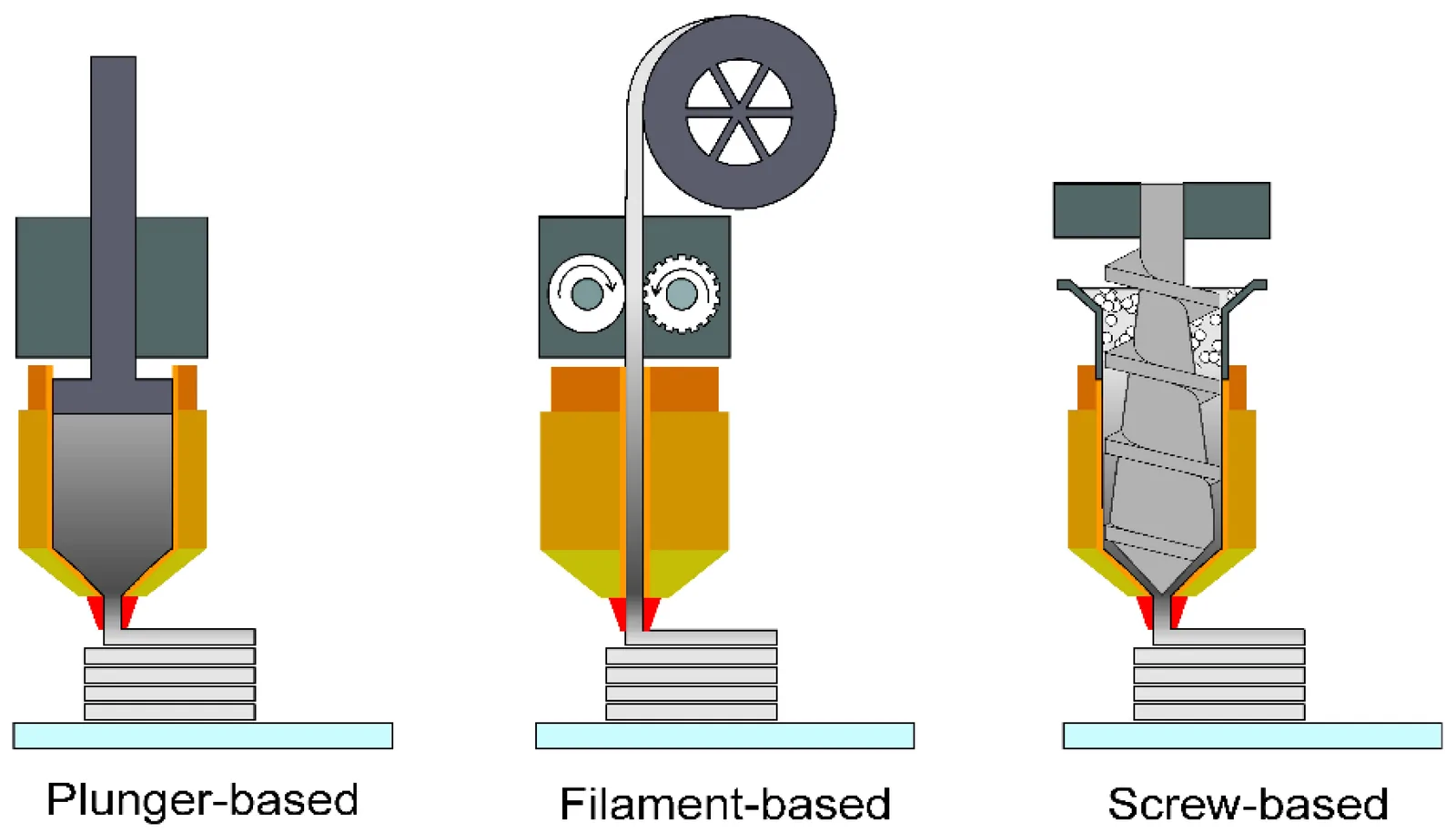
Different types of and approaches to extrusion-based additive manufacturing. Reproduced with permission from [39].

Material extrusion, often referred to as FDM or FFF, originated as a proprietary technology from Stratasys, and its patent expiration in 2009 catalyzed the consumer 3D-printing surge; the method continues to operate on the fundamental principle of a heated extruder dispensing molten thermoplastic filament in layers along a computer-governed trajectory [33]. The quality of the print output in this procedure is contingent upon a network of interrelated factors, such as infill configuration and density, nozzle and bed temperatures, nozzle diameter, build orientation, air gap, raster width and angle, and layer thickness [34]. Previous studies have addressed these through modifications in design and real-time monitoring techniques [35]. In addition to single-material printing, an increasing amount of research has advanced FDM toward multimaterial and multi-color functionalities, employing techniques such as multi-nozzle extruder heads, specialized nozzle designs for filament blending at junctions, and integrated conductive filaments for functional applications, although the boundary interfaces between different materials continue to be a significant mechanical vulnerability [36]. Simultaneously, pellet-based extrusion has surfaced as a substitute for filament feeding, enabling printers to utilize raw granules rather than pre-formed filament. This facilitates the use of materials that are challenging or unfeasible to transform into filament and significantly reduces material expenses. Also, it accommodates a range from compact benchtop systems to large-scale, robotic-arm-operated printers capable of constructing substantial structures from bulk polymer or composite pellets [37,38]. However, in both filament- and pellet-based methods, the primary engineering obstacles persist. These include ensuring stable and precisely regulated melt flow, reducing process flaws such as blockages or inconsistent extrusion, and enhancing multimaterial integration, all of which continue to influence current research and commercial advancements in material extrusion additive manufacturing [33]. Figure 3 shows the different types of and approaches to extrusion-based additive manufacturing.

### 2.3. Selective Laser Sintering (SLS)

Selective laser sintering builds three-dimensional parts by using a laser to selectively heat and fuse powdered material, layer by layer, across a powder bed. Specifically, a spreading system lays down each thin layer, the laser traces the required cross-section based on the digital CAD design, and the loose surrounding powder simply supports the structure as it forms [40]. This means that no separate support scaffolding is needed and the finished parts are later dug out or sieved from the powder bin once cooled. As a result, achieving a good result from the process involves a fairly long list of parameters [41]. In particular, laser power and scan speed together set the “energy density” delivered to the powder, and both are closely tied to hatch spacing and layer thickness, with thinner layers, tighter hatch spacing, and moderate-to-higher laser power generally producing smoother surfaces, higher density, and better mechanical strength, while excess energy or overly coarse settings lead to warping, porosity, or poorly fused layers. Bed and preheat temperature matter just as much: keeping the powder bed close to the material’s melting point reduces thermal gradients and warping, and the feed powder is normally held somewhat cooler than the build bed to stop it from prematurely clumping [42]. The powder itself is just as influential as the machine settings, since particle size, shape, and size distribution affect flowability, packing density, and how evenly a layer sinters. Fine, spherical, narrowly-distributed particles producing the smoothest, most consistent parts, which is why nylon-based powders (PA11, PA12) dominate commercial SLS, alongside other options such as polypropylene, PEEK, and TPU, depending on the strength, flexibility, or thermal performance required [43]. These material and process controls translate not only into the advantages of SLS, which include durable and geometrically complex parts without tooling, but also its recurring limitations, which include relatively rough as-printed surfaces, powder degradation on reuse, residual thermal stresses, and labor-intensive post-processing to recover and clean parts [44].

## 3. Pre-Printing Modification Strategies

Modification prior to printing refers to changes applied at the feedstock level, where modifications are applied to the polymer matrix, resin, or ink composition rather than to the surface of an already printed object. Such modifications are usually achieved by adding functional fillers, nanoparticles, and additives within the material being printed so that the functionality is integrated into the bulk material rather than just an exposed surface. Compared to post-printing methods, this approach allows for a more even distribution of the active sites and better mechanical interfacial properties between the functional material and the host material, although it may have detrimental effects on printability and the exposure of the active sites.

### 3.1. Composite Filament Fabrication (FDM)

Composite filament fabrication is the most common pre-printing process used for FDM, which entails blending and extruding functional additives such as metal oxides, carbonaceous compounds, or biochars into the thermoplastic material before the formation of filaments. This results in the creation of a printing-ready material with functionality incorporated across its whole cross-section, but filler content is always limited by the requirements of extrudability and uniform filament diameter.

**Figure 4:**
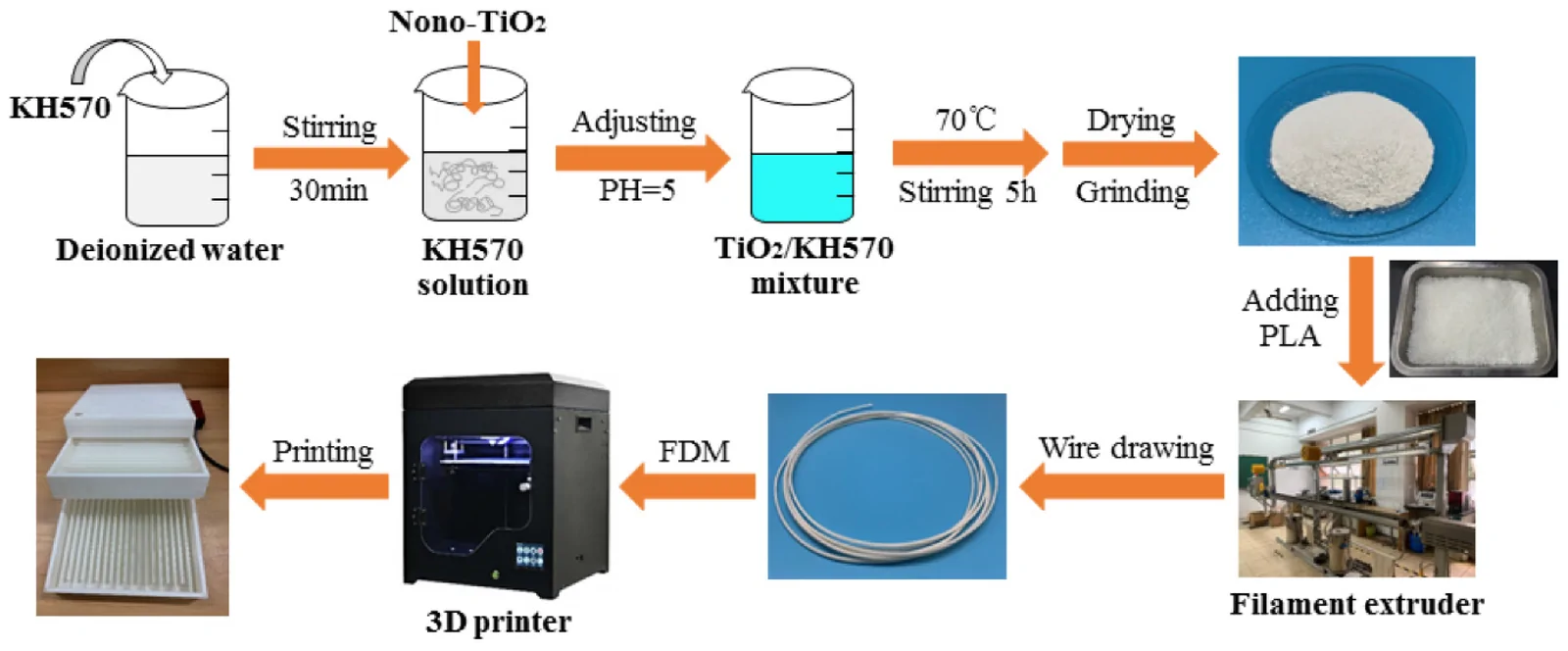
Modified TiO_2_ preparation process, material production, and finished product printing process. Reproduced with permission from [45].

Mai et al. used FDM 3D printing to fabricate a PLA-based photocatalytic water-treatment unit, first pre-modifying nano-TiO_2_ with a silane coupling agent (KH-570) to improve its dispersion within the PLA filament, then post-coating the printed units with a UV-curable, silver-loaded TiO_2_ photoresin to enhance surface photocatalytic activity [45]. Figure 4 shows the preparation of this composite. The system was tested for photocatalytic degradation of methylene blue (MB) and photocatalytic disinfection of *E. coli* under UV irradiation. FTIR and XRD confirmed successful silane grafting and TiO_2_ incorporation, while TGA showed that silane-modified TiO_2_ improved the composite’s thermal stability. Degradation efficiency scaled with the number of catalytic units connected in series (reaching 63% photocatalytic MB decolorization with four units) and with UV intensity (up to 79% at higher irradiance), and the silver-loaded TiO_2_ coating outperformed the uncoated composite for both dye degradation and *E. coli* inactivation; this is attributed to silver suppressing photogenerated carrier recombination and directly disrupting bacterial enzyme activity. Notably, the coating also appeared to protect the underlying PLA substrate from UV-induced photodegradation, addressing a key durability concern for polymer-based printed reactors.

Another study utilized FDM 3D printing, where microalgae biomass *Chlorella pyrenoidosa* (Cp) was pre-blended into a PLA-Poly(butylene adipate-co-terephthalate) (PBAT) matrix via twin-screw melt extrusion before being drawn into filament and printed into scaffolds (PIBCPx) for MB dye removal [46]. Rheology and mechanical testing showed that Cp acted as a plasticizer at low loadings but reduced tensile and impact strength at higher content due to poor PLA/PBAT-Cp compatibility, though PIBCP30 retained mechanical properties suitable for practical use. Characterizations via SEM/TEM confirmed that by increasing the Cp content, scaffold porosity and roughness were raised, with microalgae fragments well dispersed in the matrix. In addition, adsorption performance improved with Cp loading (92.66% MB removal for PIBCP30 vs. 0.06% for the unmodified scaffold), followed pseudo-second-order kinetics and the Langmuir isotherm, and was spontaneous/endothermic. FTIR also confirmed that Cp’s functional groups remained intact after the extrusion/printing process and mediated MB binding via electrostatic interaction and hydrogen bonding. Notably, reusability testing over six adsorption–desorption cycles (HCl-based desorption) retained 72% of the original capacity, supporting the scaffold’s potential for large-scale reuse.

Fijoł et al. used pre-modified filaments, in which PLA was reinforced with either TEMPO-oxidized cellulose nanofibers (TCNF) or chitin nanofibers (ChNF, 5 wt%) via physical blending and thermally induced phase separation (TIPS) pelletization prior to filament extrusion [47]. They then printed the filaments into cylindrical and hourglass-shaped filters with uniform or gradient porosity for both Cu(II) adsorption and microplastics removal from real laundry effluent. Both nanofiber composites dramatically outperformed reference PLA filters for copper adsorption (up to 234 mg/g-NF for TCNF/PLA vs. 4–10 mg/g for plain PLA at 10 mM), which is attributed to carboxyl groups (TCNF) and amine/amide groups (ChNF) enabling electrostatic/chelation binding. Mechanical testing revealed that gradient-pore designs enhanced compressive modulus and toughness far more than uniform-pore designs (up to 117% higher energy dissipation for gradient ChNF/PLA), highlighting print architecture as a tunable design lever independent of material choice. Notably, 3D printing itself induced significant pore shrinkage (up to 42%) relative to CAD-programmed dimensions, with nanofiber-loaded filaments shrinking more than pure PLA. Recyclability was limited for metal adsorption (efficiency dropped ~50% after cycle one), but the spent filters were reused for microplastics separation via size exclusion (up to 54% removal, reusable ~10 times), proposing a sequential “adsorb-then-filter” life-cycle strategy for the same device.

### 3.2. Functional Ink and Paste Formulation (DIW)

Direct ink writing is dependent on formulating functional pastes or inks where active fillers such as metal oxides, zeolites, or carbon-containing materials are suspended in a viscoelastic carrier at optimized loadings and rheology, so that shape retention can be guaranteed during extrusion. Direct ink writing provides more freedom in choosing fillers and the combination of materials as compared to filament-based printing techniques because the printability is defined by the paste rheology rather than the mechanical limitations associated with a solid filament.

Gonçalves et al. used DIW to 3D-print geopolymer lattices formulated in situ from 50 wt% red mud (RM), a bauxite residue, blended with metakaolin (MK) and an alkaline activator (with PEG added as a rheology modifier), producing ordered, mesoporous 3D-50RM structures for MB removal [48]. In this way, they succeeded in valorizing a hazardous industrial waste directly into the printed material rather than as a post-loaded additive. Despite having a lower specific surface area than a pure metakaolin control (55 vs. 97 m^2^/g), the RM-containing lattice still achieved strong performance (qe = 19.96 mg/g, among the best reported for porous geopolymer adsorbents), best described by the Langmuir isotherm and pseudo-second-order kinetic models, with uptake attributed to electrostatic and hydrogen-bonding interactions enhanced by alkali leaching that raised the solution pH over time. Leaching tests confirmed successful immobilization of RM’s hazardous elements (Cr, Cu, Pb) over 28 days, addressing a key environmental safety concern for waste-derived adsorbents. Notably, the lattice maintained strong performance over ten adsorption/thermal-regeneration cycles (cumulative uptake 95.1 mg/g) with no structural cracking, and continuous-flow column testing achieved ~90% removal within 3 h-together with the mechanical strength retained despite RM’s lower reactivity (10.66 MPa vs. 17.31 MPa for the MK-only control), supporting feasibility for industrial-scale, circular-economy wastewater treatment.

In a similar study, PVDF-based filters were loaded with powdered activated carbon (PAC) as the active adsorbent, with PVP polymers incorporated as pore-forming/hydrophilizing agents [49]. After printing, the filters underwent non-solvent-induced phase separation (NIPS) in a water bath to generate internal porosity, targeting MB removal. Contact angle and BET measurements confirmed PVP’s critical role in hydrophilizing the otherwise hydrophobic PVDF/PAC matrix (with the surface area increasing from 382 to 428 m^2^/g after phase inversion). This hydrophilicity proved essential functionally: filters without PVP achieved only 18.6% MB removal versus 93.4% with PVP included—a 402% improvement. The filter achieved a strong maximum capacity of 167.89 mg/g, with pseudo-first-order kinetics suggesting that physisorption dominated. Notably, adsorption performance was largely pH-independent (93–96% efficiency across pH 4–10, aided by a consistently negative zeta potential of −28 to −38 mV) and held up well when tested against real spiked seawater and freshwater samples (81–95% efficiency). The filter also demonstrated a simple stacking strategy to boost capacity for higher pollutant loads, and while boiling-water regeneration allowed for reuse, efficiency dropped substantially by the third cycle due to incomplete MB desorption—notably, without any structural damage or PAC leaching from the printed matrix, which is an advantage over conventional packed-bed PAC systems.

Almeida et al. formulated 3D-printed geopolymer lattices from a dual-waste precursor system-red mud (RM, fixed at 40 wt%) combined with pulp-and-paper fly ash (FA, substituting metakaolin at 0–40 wt%), systematically incorporating up to 80 wt% total industrial residues [50]. Then, printing parameters (nozzle diameter, filament rotation angle) were optimized before evaluating the structures for continuous-flow Pb(II) adsorption. Calorimetry showed FA substitution reduced geopolymerization heat release (lower reactivity than metakaolin), yet compressive strength actually peaked at 30 wt% FA (26.9 MPa), exceeding all comparable literature values for waste-based printed geopolymers-due to a compensating lower liquid-to-solid ratio required for printable FA-containing inks. Design parameters proved impactful: reducing nozzle diameter and using 45° (vs. 90°) filament rotation increased surface area and porosity but decreased compressive strength, while 90° rotation gave superior mechanical performance via better vertical filament alignment. In continuous-flow Pb adsorption tests, 45°-rotated structures showed faster initial removal (43% within 15 min, attributed to their higher surface area) though both geometries converged to >95% removal by 2 h. Leaching tests under acidic flow conditions (pH 4, 8 h) confirmed that hazardous trace elements (Zn, Cu, Ni, As) from both RM and FA remained largely immobilized at non-concerning levels, supporting the feasibility of this dual-waste valorization strategy for water treatment.

### 3.3. Resin Doping (SLA/DLP)

In resin doping, it is important to distribute the fillers or nanoparticles into the photocurable resin before it is printed, such that the active material is incorporated into the polymer matrix due to UV or visible-light irradiation. This technique allows for the creation of small-scale features, but due to the opaque and light-absorbing nature of the fillers, their loading level can be limited.

Asava-arunotai et al. used stereolithography to print a buoyant, fin-shaped polymeric structure doped with Sr-doped TiO_2_ photocatalyst and biowaste-derived silica adsorbent, designed for combined adsorption–photocatalysis removal of MB [51]. Characterisations through XRD and Raman confirmed a mixed anatase-brookite TiO_2_ phase, while XANES and PL spectroscopy showed that Sr doping introduced oxygen vacancies that suppressed charge-carrier recombination. Notably, sugarcane-leaf-derived silica (168 m^2^/g) far exceeded bagasse ash (8.5 m^2^/g) in terms of surface area and adhered more uniformly to the printed structure, translating directly into superior device performance: the sugarcane-SiO_2_/TiO_2_ structure achieved 84.51% total MB removal (48% adsorption + ~17% photocatalysis) versus 64.33% for the bagasse-ash version. The buoyant fin design was a deliberate engineering solution enabling the structure to float and be easily retrieved, thereby directly addressing the powder-recovery problem that limits standalone photocatalyst nanoparticles. Reusability over 10 cycles showed only a 12% efficiency drop, demonstrating strong operational stability for both adsorption and photocatalytic components.

Wang et al. also used stereolithography to print face-centered cubic structures (FCCSs) from a photocurable resin pre-doped with commercial TiO_2_ nanoparticles (0.2–2 wt%, eight types spanning 5–100 nm), targeting As(III) removal from water [52]. Doping concentration and nanoparticle size proved critical trade-offs: higher TiO_2_ loading improved removal efficiency but increased particle aggregation and reduced mechanical integrity (tensile strength dropped from 19.4 to 9.9 MPa), while smaller nanoparticles (5–10 nm) generally outperformed larger ones (100 nm), though one mid-sized variant (AL25) unexpectedly underperformed, which is attributed to fewer intrinsic active sites. Interestingly, embedding TiO_2_ in the printed matrix sometimes enhanced As(III) capacity relative to the free nanoparticles and sometimes reduced it, since printing allowed nanoparticles be configured into higher-surface-area macrostructures but also buried some particles’ active sites within the resin. Adsorption fit both pseudo-first-order kinetics and Freundlich isotherm well, and the optimized FCCS achieved >96% As(III) removal within 24 h, retained performance over 10 NaOH-regeneration cycles, and successfully treated real contaminated groundwater (33–53% removal), though performance there was reduced by competing ions, demonstrating a key practical advantage of eliminating post-filtration/particle-recovery steps needed for free nanopowders.

Another interesting study prepared an interconnected cube ceramic scaffold that was post-treated by dip-coating in PVDF/DMF solution and calcinated under N_2_ atmosphere to convert the polymer into microporous PVDF-derived carbon coating (840 m^2^/g) on the ceramic surface to extract Bisphenol A (BPA) and other organic pollutants [53]. The bare ceramic scaffold and PVDF-coated ceramic scaffold showed no measurable BPA removal, indicating that the carbonized polymer rather than the support material or precursor polymer was responsible for the activity. The carbonized material showed 100% BPA removal within 2 h, pseudo-second-order kinetics and a Langmuir isotherm fit with a maximum adsorption capacity of 122.5 mg/g. The developed device exhibited high versatility by showing near 100% removal efficiency for six other pollutants, both separately and as a mixture, and worked well in real water samples, including groundwater and sewage treatment plant water samples with nearly 99% removal of the pollutants. Reusability was also high by maintaining over 90% of the initial removal efficiency after 10 cycles and even the ceramic support material could be reused by burning off the old carbon layer. Table 2 summarizes various materials that underwent modification prior to 3D printing.

Regarding reusability, the pre-printing modified adsorbents showed considerable differences that depended mainly on the printed matrix and the regeneration method it could tolerate. Inorganic and ceramic-based structures were the most robust, as the red-mud geopolymer lattices maintained their performance over ten thermal-regeneration cycles [48], the TiO_2_-doped resin structures retained their performance over ten NaOH-regeneration cycles [52], and the carbon-coated ceramic scaffold retained more than 90% of its initial efficiency after ten cycles [53]. Similarly, the Sr-doped TiO_2_ photopolymer structure lost only 12% of its efficiency after ten cycles [51]. In contrast, thermoplastic-based adsorbents, which cannot withstand harsh regeneration conditions, were less stable. The microalgae-loaded PLA/PBAT scaffold retained 72% of its capacity after six cycles [46], the efficiency of the cellulose-reinforced PLA filters dropped by about 50% after the first cycle [47], and the PVDF/PAC filters lost much of their efficiency by the third cycle due to incomplete desorption in boiling water, although no structural damage or PAC leaching was observed [49]. These results indicate that embedding the active phase protects it from detachment, but capacity retention is largely determined by the regeneration conditions that the printed matrix can withstand.

Only a few pre-printing modified adsorbents were tested in real water matrices. The PVDF/PAC filters retained 81–95% of their efficiency in spiked seawater and freshwater [49], and the PVDF-derived carbon-coated scaffolds achieved nearly 99% removal in groundwater and sewage treatment plant effluent [53], whereas the TiO_2_-doped structures removed only 33–53% of As(III) from real groundwater owing to competing ions [52]. In geopolymers, alkali leaching can also raise the solution pH and thus affect adsorption [48], although their hazardous elements remained immobilized even under acidic flow [50]. Therefore, matrix effects should be assessed systematically rather than only in single-pollutant solutions.

Regarding selectivity, a distinction should be made between selectivity demonstrated experimentally and selectivity inferred from the properties of the adsorbent. Among the pre-printing modified adsorbents, selectivity was confirmed in competitive multi-ion tests only for a limited number of alginate- and GO-based inks, such as the CA/GO structures, which showed 99.7–99.95% selectivity for Pb(II) or U(VI) over several coexisting metals [54,55,56], the DMSA-GO/cellulose and mercapto-GO/chitosan inks, which preferentially adsorbed Cu(II) [57], and the PEI/GO/alginate adsorbent, which selectively recovered Au(III) from real e-waste leachate [58]. For the remaining studies, which were tested only in single-pollutant solutions, selectivity can only be inferred from the functional groups or surface charge of the filler and should therefore be treated with caution. As for continuous-flow operation, only the geopolymer lattices were tested beyond batch conditions. The red-mud/metakaolin lattice removed about 90% of MB within 3 h in a column [48], while in the red-mud/fly ash lattices the 45° filament orientation yielded faster initial Pb(II) removal than the 90° one (43% within 15 min), although both exceeded 95% after 2 h [50]. Breakthrough curves, pressure drop and bed utilization were not reported in detail, so the batch capacities of pre-printing modified adsorbents cannot yet be translated into column performance.

### 3.4. Printing Parameters

The adsorption performance of a 3D-printed adsorbent depends not only on the active material but also on the architecture created during printing. Unlike conventional shaping methods, 3D printing allows for close control over scaffold geometry, such as channel size, cell density and wall thickness, as well as over properties like hierarchical porosity and mechanical strength [59]. In extrusion-based printing, this architecture is set mainly by the so-called structural parameters, namely, layer thickness, infill density, infill geometry, raster angle and raster gap [60]. Because adjacent filaments solidify before they fully fuse, printed parts always contain voids and partly bonded interfaces [36]. As a result, the printing parameters are directly linked to the porosity and anisotropy of the final structure [61]. Pore size, in particular, acts at two levels. At the macroscale, open printed geometries reduce flow resistance and improve mass transfer. For example, 3D-printed zeolite 13X monoliths reached a CO_2_ capacity similar to that of commercial beads (3.5 vs. 3.3 mmol/g) while showing sharper breakthrough fronts and therefore faster kinetics [62]. Making the channels too small, however, can have the opposite effect. ZIF-8 monoliths with 250 µm cells gave broader breakthrough fronts than those with 600 µm cells, because the uneven flow did not leave enough contact time for the adsorbate to reach the walls [59]. At the microscale, porosity inside the walls is just as important, since creating macropores with porous binders or by burning out the plasticizer greatly improved mass transfer [59]. In FDM printing, the openings between deposited filaments are controlled by the air gap: positive gaps produce loosely packed, more open structures, whereas negative gaps produce denser structures with lower porosity [36].

Infill density and layer thickness also play a key role, mainly by controlling how dense the printed walls are. Infill density can range from a hollow part (0%) to a fully solid one (100%) [60], and in adsorbent scaffolds it corresponds roughly to cell density and void fraction [59]. A higher cell density increases the geometric surface area per unit volume and can improve film mass transfer. This holds, however, only when the walls are thin or porous enough [59]. When zeolite 13X monoliths were printed at 200, 400 and 600 cpsi (cells per square inch), mass transfer actually became slower as cell density increased. The denser structure restricted flow and did not allow enough time for the adsorbate to diffuse into the walls. On the other hand, denser structures are mechanically stronger. For instance, the tensile strength of PLA rose from 17 to 27 MPa when the infill increased from 35% to 100% [36], and adequate strength is needed for the scaffold to withstand flow and repeated use [59]. Layer thickness, typically 0.17–0.33 mm [61], or about 25–75% of the nozzle diameter [36], acts in a similar way. Thicker layers leave more voids and less contact between filaments, whereas thinner layers produce denser, better-bonded structures with smoother surfaces [36]. This matters for adsorption because printed scaffolds usually have thicker walls than extruded monoliths, so their mass transfer is mostly controlled by diffusion within the walls rather than by transfer from the fluid to the surface [59]. Consequently, thick and dense deposits can slow down adsorption kinetics, even when the overall capacity is retained.

Finally, the lattice geometry, or infill pattern, determines how the fluid moves through the adsorbent. FDM offers many patterns, including rectilinear, grid, triangular, honeycomb, gyroid and cubic [61]. Their influence on mechanical behavior is small at high infill but much less understood at low infill [36], which is precisely the range of interest for porous adsorbents. Compared with the straight, separate channels of extruded monoliths, 3D printing can create interlocking and three-dimensionally varying channels. Computational fluid dynamics simulations have shown that such geometries improve contact between the fluid and the solid. The trade-off is a higher pressure drop (207 vs. 6.9 Pa), which nevertheless remains far below that of packed pellets. Similarly, monoliths printed with 30°, 45°, 60° and 90° channel offsets outperformed an extruded monolith, while wall-less designs improved contact further, again at the cost of a higher pressure drop [59]. It should also be noted that these parameters strongly interact with each other, so optimizing them one at a time does not lead to the same result as optimizing them together. For this reason, multivariate approaches such as design of experiments are recommended [36]. Overall, designing a printed adsorbent requires a balance between an open, porous architecture that favors mass transfer, an acceptable pressure drop, and enough mechanical strength for repeated use.

**Table 2:** Summary of pre-printing modifications on adsorbent materials.

Name	3D Printing Technology	Type of Modification	Pollutant	Adsorption Conditions	Qm (mg/g)	Regeneration	Selectivity	Ref.
MOF(MIL-88A)/MoS_2_–alginate/CMC/gelatin aerogel	DIW (extrusion) + freeze-drying	MOF/MoS_2_ heterojunction pre-mixed into hydrogel ink (5 and 7.5 wt%)	Methylene blue (MB)	Dosage: 4–6 g/L pH: n.r. * Initial concentration: 5 mg/L contact time: 1440 min	2.04	5 cycles; 34–42% efficiency loss (combined adsorption/photocatalysis reuse)	Not tested	[63]
PLA/PBAT/Chlorella pyrenoidosa (PIBCPx)	FDM	Microalgae biomass (Cp, up to 30 phr) melt-blended into PLA/PBAT filament	Methylene blue (MB)	Dosage: n.r. pH: n.r. Initial concentration: 100 mg/L contact time: 1440 min	43.80	6 cycles; 72% capacity retained	Not tested	[46]
TCNF/PLA and ChNF/PLA biocomposite filters	FDM	TEMPO-oxidized cellulose or chitin nanofibers (5 wt%) blended into PLA via TIPS pelletization	Cu(II) (+microplastics by size exclusion)	Dosage: 18.75–31.25 g/L pH: 6.0–7.5 Initial concentration: 63.5 and 635.5 mg/L contact time: 480 min	234.00	Efficiency drops ~50% after cycle 1; filters repurposed for microplastics after Cu desorption	Not tested	[47]
3D-printed red-mud/metakaolin geopolymer (3D-50RM)	DIW (robocasting)	Red mud (50 wt%) formulated directly into the geopolymer ink with metakaolin/PEG	Methylene blue (MB)	Dosage: 3 g/L pH: 5.6–7.3 Initial concentration: 100–200 mg/L contact time: 1440 min	19.96	10 cycles (thermal, 400 °C); cumulative uptake 95.1 mg/g, no significant drop	Not tested	[48]
PVDF/PAC 3D-printed filter	DIW	Powdered activated carbon mixed into PVDF/PVP paste; porosity generated by phase inversion (NIPS)	Methylene blue (MB)	Dosage: n.r. pH: 4–10 Initial concentration: 200 mg/L contact time: 480 min	167.89	Boiling-water regeneration; stable for 2 cycles, drop by cycle 3	Not tested; real-matrix performance only (81–95% retained in seawater/freshwater)	[49]
Sr-doped TiO_2_/biowaste-SiO_2_/polymer structure	SLA	Sr-doped TiO_2_ photocatalyst + biowaste-derived silica (sugarcane leaves or bagasse ash) pre-mixed into photocurable resin	Methylene blue (MB)	Dosage: n.r. pH: n.r. Initial concentration: n.r. contact time: 1440 min (dark)	n.r.	10 cycles; >70% retained (12% drop, not statistically significant)	Not tested	[51]
TiO_2_ nanoparticle-doped FCCS lattice	SLA	Commercial TiO_2_ NPs (0.2–2 wt%, various sizes) doped into photocurable resin	As(III)	Dosage: n.r. pH: 7 Initial concentration: 0.1 mg/L contact time: 1440 min	2.83	10 cycles (NaOH regeneration); no significant decrease	Not tested; real-matrix performance only (33–53% removal in groundwater, reduced by competing ions)	[52]
DMSA-modified GO/cellulose (DMSA-GO/CE)	DIW	DMSA-functionalized GO pre-dispersed into a cellulose/sodium alginate printing ink	Cu(II)	Dosage: 0.4 pH: 5 Initial concentration: 20 mg/L contact time: 90 min	270.00	5 cycles (HCl); ~80% retained (46.96 mg/g)	Selective for Cu over Pb, Cr, Cd (electronegativity-driven)	[57]
BR-GO-DETA/BR-AC-DETA Gyroid composite	SLA (vat photopolymerization)	GO or activated carbon (0.5 wt%) pre-dispersed in the photocurable resin (pre-print component)	Diclofenac	Dosage: 1 g/L pH: 7 Initial concentration: 20 mg/L contact time: 1440 min	91.50 (GO); 12.30 (AC)	5 cycles (GO)/3 cycles (AC); 80–98% retained after cycle 2	Not directly tested; ~20% loss in real pharmaceutical wastewater	[64]
3D CA/GO (calcium alginate/graphene oxide) porous adsorbent	DIW	GO pre-mixed 1:1 (*v*/*v*) into sodium alginate ink (no reduction step), Ca^2+^-crosslinked	Pb(II)	Dosage: 0.4 g/L pH: 3.0 Initial concentration: 100 mg/L contact time: 1440 min	490.20	8 cycles (HCl elution); only 8.26% capacity drop	99.8% selective for Pb(II) over Cr, Cd, Ni, Hg, Cu, Co, Zn, As	[55]
Alginate/Nafion/rice-husk biochar hydrogel (Alg/Biochar10)	DIW (bioprinter)	Hydrothermally-produced rice-husk biochar (1–10 wt%) pre-mixed into alginate/Nafion ink	Ibuprofen (IBU); Methylene blue (MB)	Dosage: n.r. pH: 7 Initial concentration: 12 mg/L each contact time: 30 min	202.20 (IBU); 233.40 (MB)	20 cycles (methanol/water desorption); <10% loss	MB preferred over IBU (63%/37% share in equimolar binary mixture)	[65]
3D CA/GO (calcium alginate/graphene oxide) porous adsorbent	DIW	GO pre-mixed into sodium alginate ink (no reduction step), Ca^2+^-crosslinked	U(VI)	Dosage: 0.4 pH: 2.8 Initial concentration: 135 mg/L contact time: 30 min	200.10	8 cycles; only 4.4% capacity drop	99.7% selective for U(VI) over As, Cd, Co, Cr, Cu, Hg, Ni, Pb	[56]
3D CGx (calcium alginate/GO, graded SA ratio, CG6 selected)	DIW	GO pre-mixed into graded-concentration sodium alginate ink (SG3–SG8), Ca^2+^ crosslinked	U(VI)	Dosage: 0.4 g/L pH: 5.0 Initial concentration: 100 mg/L contact time: 2880 min	740.74	9 cycles; only 2.3% capacity drop	99.95% selective for U(VI) over Cd, Co, Fe, Mn, Pb, Sb, Zn	[54]
PCL/sodium alginate filaments and 3D biomimetic structures	3D pen/FDM/“growing robot” tip-extrusion	Sodium alginate (5–30 wt%) pre-blended into PCL/DCM solution before heat-extrusion into filament	Cu(II)	Dosage: 16.7 g/L pH: n.r. Initial concentration: 100 contact time: 43,200 min	93.30 (filament); 22–24 (3D structures)	Not tested	Not tested	[66]
3D CA (pure calcium alginate) porous adsorbent	DIW	Sodium alginate ink (no filler), Ca^2+^-crosslinked	U(VI)	Dosage: 0.4 g/L pH: 2.5 Initial concentration: 167 mg/L contact time: 1200 min	117.30 (pH 2.5); 410.1 (pH 7.4)	6 cycles (HCl elution); ~18% capacity drop	24% U(VI) selectivity vs. Na, Co, Cs, Sr in simulated radioactive wastewater (highest of the set)	[67]
3D-PA@Bent (polyamide-12/Na-bentonite)	SLS	Na-activated bentonite clay (40 wt%) pre-blended with PA12 powder before laser sintering	Sr(II)	Dosage: pH: 7.7 Initial concentration: contact time:	10.60	Not tested	Sr > Ca > Mg > K ≫ Na (synthetic); 22.7% Sr removal in real mining wastewater	[68]
SGCS-E3 (mercapto-GO/chitosan/ε-polylysine)	DIW	Thiol-functionalized GO, chitosan, and antimicrobial ε-polylysine pre-mixed into ink with sacrificial SiO_2_ template	Cu(II) (+antimicrobial/antifouling function)	Dosage: 34 g/L pH: 5 Initial concentration: 20 mg/L contact time: 1440 min	260–431	10 cycles (HCl); >80% retained	Selective for Cu(II) in mixed-ion competitive test	[69]
Cu-BTC(HKUST-1)/calcium-alginate-gelatin (hexagon pattern)	DIW	Pre-synthesized, PVP-capped Cu-BTC nanoparticles (up to 13 wt%) mixed into SA-gelatin ink, Ca^2+^-crosslinked	Methylene blue (+MV, MG, RB, AO)	Dosage: 1 g/L pH: 6.4 Initial concentration: 20 mg/L contact time: 20 min	Not reported	7–10 cycles; 64–82% retained depending on print pattern	Not tested; broad-spectrum (non-selective) removal across 5 dyes and 1 L pilot scale	[70]
DGSP-1 (dual-network GO/sodium-alginate/PVA aerogel)	DIW	GO and PVA pre-mixed into sodium alginate ink; PVA freeze-thaw crosslinked plus Ca^2+^ ionic network	Cu(II)	Dosage: 1 g/L pH: 5 Initial concentration: 20 mg/L contact time: 180 min	208.00	5 cycles (HCl); 71.5% retained	Cu(II) and Pb(II) adsorbed better than other competing metals	[71]
3D PGC (polyethyleneimine/GO/calcium-alginate)	DIW	PEI and GO pre-mixed into sodium alginate ink; dual crosslinked post-print (CaCl_2_ + glutaraldehyde covalently anchoring PEI)	Au(III)	Dosage: 0.2 g/L pH: 2.5 Initial concentration: 100 mg/L contact time: 1320 min	1057.00–1527.00	8 cycles; no significant decrease	57.4% Au(III) selectivity vs. Zn, Pb, Ni, Cu, Co, Cd (11.7–44.9%); 96% efficiency on real e-waste leachate	[58]
GHA (coaxial hollow-filament alginate/cellulose-nanocrystal)	Coaxial DIW (core–shell extrusion)	Cellulose nanocrystals pre-mixed into alginate ink; hollow filaments co-extruded with CaCl_2_ crosslinker core	Cu(II) (+Zn(II), Cd(II), Cr(III))	Dosage: 2.47 g/L pH: 5.4 Initial concentration: 300 mg/L each contact time: 180 min	97.20–102.10	5 cycles (HCl); 88% retained	Cu(II) > Cr(III) > Zn(II) > Cd(II)	[72]

* n.r. = not reported.

## 4. Post-Printing Modification Strategies

**Figure 5:**
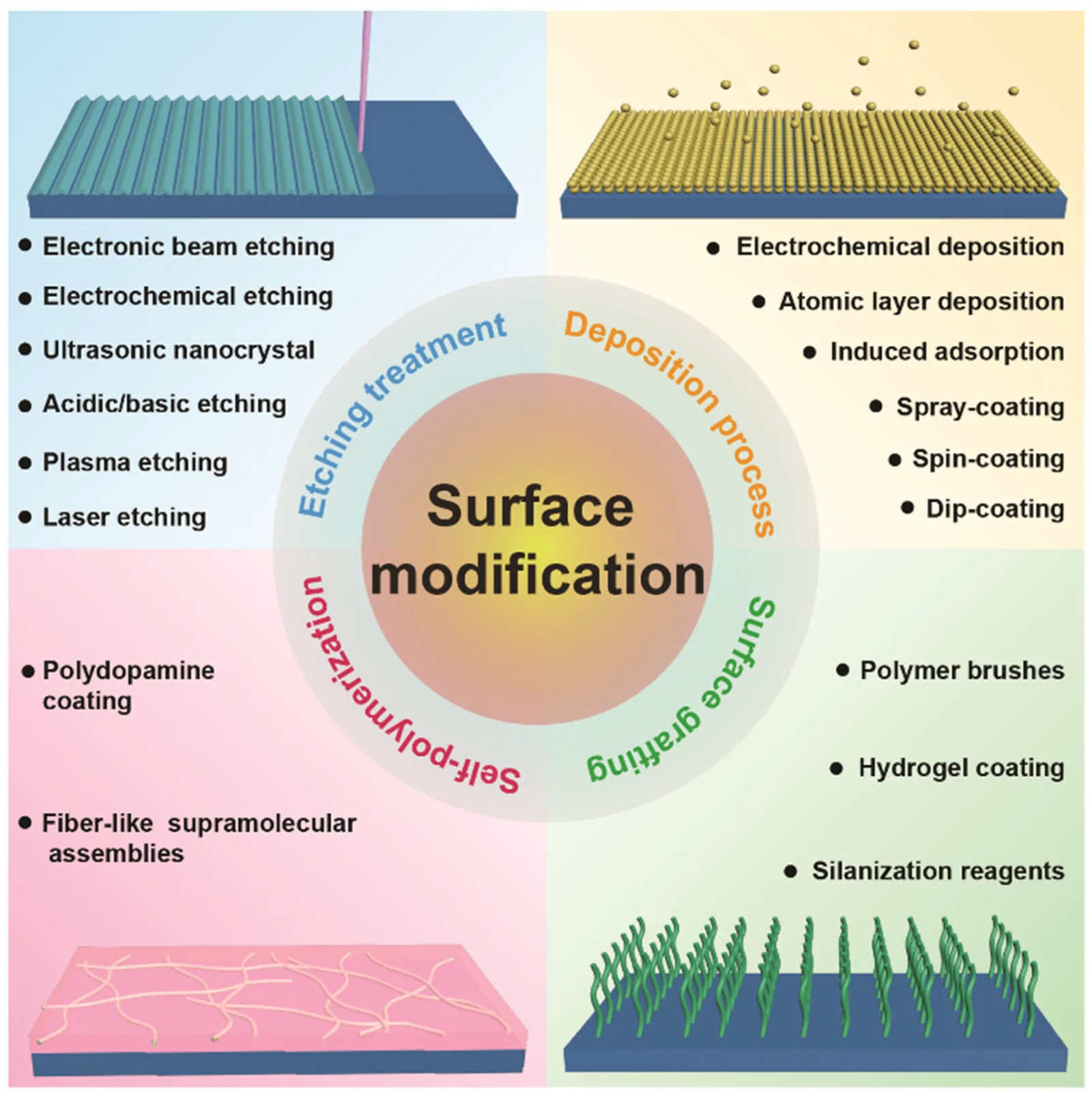
The methodologies for surface functionalization for 3D printing. Reproduced with permission from [73].

Surface modification has become an essential component of technological advancement, supporting a diverse array of practical applications in both industry and daily life. It employs multiple physical and chemical processes to create surfaces that are customized with specific functionalities. In the context of surface-functionalized 3D printing, the modification technique chosen must be compatible with the printed structure and its intended application. To achieve this, a variety of post-processing methods have been devised, as summarized in Figure 5. Water remediation is one area in which such technologies are urgently required, as organic contaminants and heavy metals continue to be significant sources of water contamination and present significant environmental and public health hazards. One major concern is that conventional wastewater treatment materials are frequently restricted by their inadequate material adaptability and poor long-term stability. Thus, surface-functionalized 3D printing provides a promising alternative, allowing for the preparation of durable, application-specific devices that are effective for water decontamination.

### 4.1. Surface Coating and Thin-Film Deposition

Surface coatings and film deposition involve applying an active layer to an already printed object by means of various methods such as dipping, spraying, electroplating, or layer-by-layer deposition to create active surfaces without affecting the whole volume of the material. This technique makes it possible to separate the process of functionalization from the process of printing since different materials can be used and optimized independently of each other. At the same time, the reliability of the coating becomes a crucial factor due to its possible delamination and wear.

Ji et al. printed a porous support layer with defined straight-through pores (300 μm, tunable porosity 20–50%), onto which a separately synthesized carboxyl-functionalized polyethersulfone (PES-COOH) was cast as an ultrathin (1–4 μm) active separation layer [74]. This resulted in the formation of a thin-film-composite forward osmosis (FO) membrane for desalination application. Carboxyl grafting (confirmed by NMR/FTIR) imparted strong hydrophilicity and a negative surface charge (−72 to −85 mV). In addition, the thinner active layers (down to 1 μm) drove pronounced surface segregation of carboxyl groups toward the water interface, simultaneously enhancing water flux and strengthening electrostatic salt rejection, breaking the typical permeability-selectivity trade-off. The optimized 1 μm-PES-COOH-TFC membrane achieved a high water flux (9.6 L/m^2^h) with very low reverse salt flux (6.3 mmol/m^2^h, selectivity 1523.8 L/mol) and maintained stable performance over 72 h of continuous operation.

Red-mud/metakaolin-geopolymer lattices were prepared using DIW, followed by multi-stage post-functionalization: catalyst-free growth of multi-walled carbon nanotubes (MWCNTs) via thermal CVD [75]. This was achieved by exploiting red mud’s intrinsic hematite as an in situ iron catalyst after H_2_ reduction. This was followed by atomic layer deposition (ALD) of a conformal anatase TiO_2_ shell onto the CNTs, targeting photocatalytic degradation of ciprofloxacin (CIP) under continuous flow. CNT growth dramatically increased the surface area to 202 m^2^/g, which is among the highest figures reported for 3D-printed inorganic polymers, and CNTs proved functionally essential, since TiO_2_-only scaffolds showed no visible-light activity and only ~50% photocatalytic CIP removal under UV after 2h, versus 92% for the CNT/TiO_2_ composite. Radical scavenger experiments revealed a hole-dominated degradation mechanism, and performance was strongly pH-dependent, tied to CIP’s protonation state and the composite’s zeta potential. The scaffold also achieved substantial mixed-pollutant removal (>90% for most of a four-drug mixture) and showed strong robustness with stable performance over five reuse cycles, with negligible Ti leaching (<0.4 mg/L) over 28 days.

In another case, PLA films were modified via in situ growth of Cu-based MOF crystals (via sequential H_3_BTC/TEA and Cu(NO_3_)_2_ solution soaks, repeated for 1–8 cycles) to create Cu-MOFs/PLA composite films for malachite green (MG) dye removal [76]. Characterization via SEM and XRD confirmed progressively denser Cu-MOF crystal coverage with increasing coating cycles (visually tracked by deepening blue color), without altering the underlying PLA structure. The eight-layer film (Cu-MOFs/PLA-8) achieved rapid, high-efficiency MG removal (>90% within just 10 min at both 5 and 10 mg/L), while bare PLA showed minimal adsorption even after 40 min, confirming the MOF coating as the sole active component. A standout feature was the dual-recyclability strategy: the MOF-coated film could be reused via acetone washing (>80% efficiency retained after three cycles, >60% after 5), and separately, the PLA scaffold itself could be fully regenerated by stripping spent MOFs with brief HCl treatment and then re-coated with fresh Cu-MOFs-effectively decoupling substrate lifespan from adsorbent lifespan. The film also performed well on real water matrices (tap, lake, and river water), achieving 90–93% MG removal.

### 4.2. Nanoparticle Decoration

Nanoparticle decoration entails the in situ growth or deposition of nanoscale active components such as metal, metal oxide, or carbon nanoparticles onto the surface of a fabricated structure by using chemical reduction, hydrothermal technique, or electrodeposition. The approach provides maximal utilization of the exposed surface of the active phase and makes it highly suitable for the fabrication of structures where surface reactivity is of primary importance. The difficulty with this approach, however, is ensuring the homogeneity of the nanoparticles and their robust adherence to the substrate, as otherwise, the nanoparticles tend to leach off during use.

In their research, Wu et al. designed five shapes of through-hole scaffolds and then post-treated them with a MOFs/MXene composite (ZIF-67/ZIF-8 in-situ grown on Ti_3_C_2_ MXene) along with polydopamine (PDA) for selective removal of Congo red (CR) dye and Hg(II) [77]. Performance was dictated by the structural design, and as such, the skew-square (having highest SSA) scaffold had a 99.13% removal efficiency for CR and 89.79% for Hg(II) as compared to just 36.93% CR and 59.53% Hg(II) removal for a triangular-shaped scaffold having the lowest SSA, with the bare scaffold making no contribution to the process. Selectivity of the MOF composite was high for anion CR dye over cation/neutral dyes and high for Hg(II) over other heavy metals. The adsorption process followed pseudo-second-order kinetics and Langmuir isotherm, with maximal capacities of 77.95 mg/g for CR and 480.95 mg/g for Hg(II), which is quite high for Hg(II) ions strongly coordinated to the adsorbent’s nitrogen/sulfur sites. XPS/FTIR revealed multi-mechanism adsorption. Finally, after 5–6 cycles of regeneration, it retained 78–82% efficiency and good mechanical integrity.

Cylindrical PLA filters were prepared via FDM technology and then pre-roughened via acetone treatment before sonication-assisted surface doping with graphene oxide (GO), targeting Cd(II) removal [78]. GO doping proved essential as XPS confirmed increased oxygen functionality after acetone treatment provided anchoring sites for GO, and kinetic tests showed that GO/PLA achieved ~14.5 higher Cd capacity than neat PLA (0.085 mg/g). Adsorption followed pseudo-second-order kinetics and the Langmuir isotherm, with a maximum capacity of 1.26 mg/g for the whole filter, but a striking 58.23 mg/g when normalized to the GO content alone (~2.16% of filter mass), highlighting how dilute the active phase was within the bulk polymer matrix. Performance was strongly pH-dependent due to GO’s increasingly negative zeta potential. Among 11 heavy metals screened at pH 6, Pb and Cd showed the highest removal (95.9% and 86.6%), correlating with electronegativity, while anionic species (Cr, As) showed negligible uptake, confirming an electrostatic-interaction-driven mechanism. Finally, in continuous fixed-bed column tests, the GO/PLA-packed column dramatically outperformed neat PLA (breakthrough time 130 h vs. 0.076 h; saturation time 1250 h vs. 15 h), with the Thomas-model-fit bed capacity (1.24 mg/g) closely matching the batch Langmuir value. This suggests that the potential for practical scalability was demonstrated, especially since HNO_3_-based regeneration retained consistent performance over cycles 2–5 despite an initial ~32% efficiency drop after the first regeneration.

### 4.3. Chemical Surface Functionalization

Surface chemical functionalization entails changing the chemistry of the surface of the printed material through chemical processes such as silanization, grafting or the addition of certain functional groups such as hydroxyl, carboxyl or amine groups, usually after activating the surface with a process such as plasma treatment or chemical etching to create reactive surfaces. This technique changes the chemical nature of the surface of the printed part without necessarily adding another layer to the surface, as would occur with coatings or nanoparticles, but forms strong covalent bonds between the functional groups and the substrate. However, the extent of functionalization that is possible is limited by the reactivity of the printed part.

A study used LCD-based stereolithography to print gyroid-shaped preceramic polymer monoliths, which were pyrolyzed at 800 °C to form silicon oxycarbide (SiOC) ceramic scaffolds [79]. Then, it was modified via silane (GLYMO) coupling and surface grafting with a chitosan-graphene oxide (chitosan–GO) composite, targeting methyl orange (MO) dye removal. BET analysis confirmed that the grafting boosted the surface area to 78.90 m^2^/g, and lower GO/chitosan ratios performed best, since excess GO diluted chitosan’s functional-group density and caused site saturation. Adsorption was strongly pH-dependent (88% removal at pH 3 vs. only 40% at pH 7, driven by amine protonation), followed pseudo-second-order kinetics and was best described by the Harkins–Jura isotherm, with a maximum uptake capacity of 182.25 mg/g at the optimal GO/C ratio. Lastly, thermodynamics indicated an endothermic process favored at higher temperatures, while NaOH-based regeneration over five cycles retained >92% removal efficiency throughout, supporting reusability.

Acetone treatment was found to be superior to NaOH treatment for achieving the etching required for GO doping in cylindrical scaffolds fabricated by FDM [80]. Additionally, no drying after treatment (0 min of drying, GO doping immediately after treatment with acetone) was the key for maximal loading of GO due to its plasticizing effect (it enhanced polymer chain mobility and surface roughness, but its effect decreased with evaporation of acetone). It had a direct effect on performance: undoped PLA removed only 1.26% of MB from the solution, while NaOH-treated PLA removed 7.07%, and the optimal acetone-treated/dried GO/PLA filter was capable of removing 74% of MB from the solution. A tensile test also proved that acetone treatment weakened PLA scaffold strength and elongation, which explained the improved GO adhesion but also showed that it decreased its strength. TGA demonstrated that GO loading was low. Adsorption kinetics followed a pseudo-second-order model, fitting the Langmuir isotherm with a maximal capacity of 4.84 mg/g.

Nurul Husna Mohd Yusoff et al. used masked stereolithography (mSLA) to print PEGDA-based monoliths, which were then post-modified via silane (GPTMS) coupling and dip-grafting with nanochitosan, followed by electron-beam (EB) irradiation after treatment to enhance surface properties, targeting methyl orange (MO) dye removal [81]. EB irradiation roughened the surface (AFM Rq rising from 0.211 μm unirradiated to 0.847 μm) without significantly altering chitosan’s chemical functionality. As a result, removal efficiency rose from 90.4% (nonirradiated) to 96% at 200 cGy, and equilibrium time dropped sharply from 2 h to just 80 min. Characterization via EDX and XPS confirmed successful chitosan/silane grafting, with the proposed mechanism combining electrostatic attraction, n-π stacking, and hydrogen bonding. The maximum adsorption capacity was 12.2 mg/g, notably lower than many other chitosan-based adsorbents, reflecting a trade-off: the monolithic format sacrifices some capacity for easy handling/recovery.

In a similar case, DLP stereolithography was used to print PEGDA monoliths, post-modified via silane (GPTMS/APTMS) coupling and dip-grafting with chitosan, and optimized using Response Surface Methodology (Box–Behnken design), targeting methyl orange (MO) dye removal alongside real textile wastewater BOD/COD reduction [82]. RSM identified pH as the dominant factor (the highest removal was at a slightly acidic/neutral pH ~4–6.3, consistent with electrostatic attraction between protonated chitosan amines and anionic MO), while dipping time showed no significant effect; optimal conditions (2 wt% chitosan, 45 min dipping, pH 6.3, 60 °C) yielded 90.4% removal. Adsorption followed the Langmuir isotherm and pseudo-second-order kinetics, with a maximum capacity of only 13.21 mg/g and an experimental value of 12.7 mg/g, which is notably modest; the authors attributed this directly to the monolith’s very small surface area, which is far below comparator chitosan adsorbents in other forms (some reaching 149–350 mg/g). Thermodynamics indicated a spontaneous, endothermic, and physically dominated adsorption process. Practically, the monolith achieved a strong 62.4% BOD reduction on real textile wastewater but only a marginal COD reduction (2%), again linked to limited surface area; reusability over four cycles retained ~78% efficiency after the second cycle, with minimal mass loss (<1%), and mechanical testing confirmed that silane grafting improved monolith stiffness.

In another study, gyroid-lattice acrylate scaffolds pre-functionalized with graphene oxide and activated carbon as the fillers through vat photopolymerization (DLP/SLA) and further functionalized using acid activation and diethylenetriamine (DETA) grafting technique were fabricated to adsorb diclofenac from water and industrial pharmaceutical wastewater [64]. Modification of the filler by DETA significantly increased wettability, whereas GO was significantly superior compared to AC. The surface area of BR-GO-DETA increased twofold by DETA grafting because of synergic pore formation, while the surface area of AC was decreased due to pore blocking, which positively affected adsorption capability (BR-GO-DETA removed ~90% DCF at pH 5 while BR-AC-DETA removed only ~30%). Adsorption was conducted following the pseudo-second-order kinetic model and Langmuir isotherm with maximum capacities of 91.5 mg/g (BR-GO-DETA) and 12.34 mg/g (BR-AC-DETA) at 30 °C. The mechanism included electrostatic attraction, hydrogen bonding, and π-π/hydrophobic interactions (confirmed by shifts in FTIR bands), and regeneration efficiency of BR-GO-DETA was 80–98% during the second cycle, showing good potential for reuse.

### 4.4. Plasma and UV Etching

Etching using plasma and UV involves changing the chemistry of the printed layer via the application of reactive ions or high-energy UV light, resulting in a physically altered roughened surface and chemical functionalization with reactive sites (such as hydroxyl or carbonyl groups). Apart from imparting chemical reactivity, etching results in roughened and more wettable surfaces, both of which may lead to increased adherence during further coating or deposition of nanoparticles. It is mainly a fast surface reaction that takes place without reagents; however, it remains shallow and therefore may be repeated in conjunction with other methods.

In a study by Wang et al., an FDM 3D-printed plain PLA lattice relied on UV irradiation for post-printing treatment (365 nm, 30 W, 10 days) as activation technique to enable the MB adsorption process [83]. The UV exposure helped increase efficiency moderately through photo-oxidation, which created oxygen-containing functional groups (hydroxyl, carboxyl) along with an increase in microporosity. As a result, the adsorption capacity at equilibrium increased from 3.56 to 4.11 mg/g, and the time to reach equilibrium decreased from 28 to 24 h. Both UV- and non-UV-treated PLA followed the pseudo-second order kinetic model and the Langmuir isotherm model, with maximum capacity reaching 5.02 mg/g (non-UV) and 5.78 mg/g (UV irradiation). From the intraparticle diffusion analysis, it became clear that the UV treatment improved the external and internal diffusion, which correlates well with the increased Langmuir affinity constant and slightly more homogeneous surface. The reusability test was conducted under five ethanol-based regenerations; it became clear that UV-treated cubes had significantly greater reusability potential compared to non-UV cubes.

### 4.5. Hybrid Modification

Hybrid modification methods consist of combinations of one or more of the methods mentioned above—for example, etching processes such as plasma etching and UV etching to form reactive sites and then decorating the resulting sites with nanoparticles or grafting molecules, or doping resins with other chemicals and subsequently coating the printed structures—in order to utilize the advantages of bulk and surface functionalization techniques. To compensate for the disadvantages associated with a specific method, such as the accessibility of active sites in pre-printing methods or the durability of surface-only modification, more stable surfaces can be obtained with hybrid methods. However, these methods are complicated by additional steps that require control.

In a study by Wu et al., a spherical, porous 3D-printed scaffold was fabricated via stereolithography was fabricated, which was subsequently post-modified by dip-coating in a chitosan/ZIF-67 (CS-ZIF-67) composite solution to produce the 3D-CS-ZIF-67 adsorbent for Pb(II) removal from aqueous solution [84]. FTIR, XRD, and FESEM/EDS confirmed successful incorporation of the ZIF-67 metal–organic framework onto the chitosan-coated scaffold surface and interior. Batch studies showed that adsorption was pH-dependent and followed pseudo-second-order kinetics and the Langmuir isotherm, with a maximum capacity of 5.47 mg/g at 30 °C, which is markedly higher than the bare 3D-printed scaffold or ZIF-67 alone, and thermodynamically spontaneous and endothermic. XPS analysis pointed to a mixed adsorption mechanism involving surface complexation with oxygen/nitrogen-containing functional groups, ion exchange between Pb(II) and Co(II) in the framework, and hydrogen-bonding/electrostatic interactions. Interestingly, in coexisting ion systems, Cu(II) enhanced Pb(II) uptake (up to ~104% increase in ternary systems) while Cd(II) suppressed it, reflecting competitive site occupancy and electronegativity differences. The composite also performed well in dynamic fixed-bed column tests (67% initial removal, exhaustion at 250 mL throughput) and, notably, required no additional separation/recovery step given its monolithic 3D-printed form.

**Figure 6:**
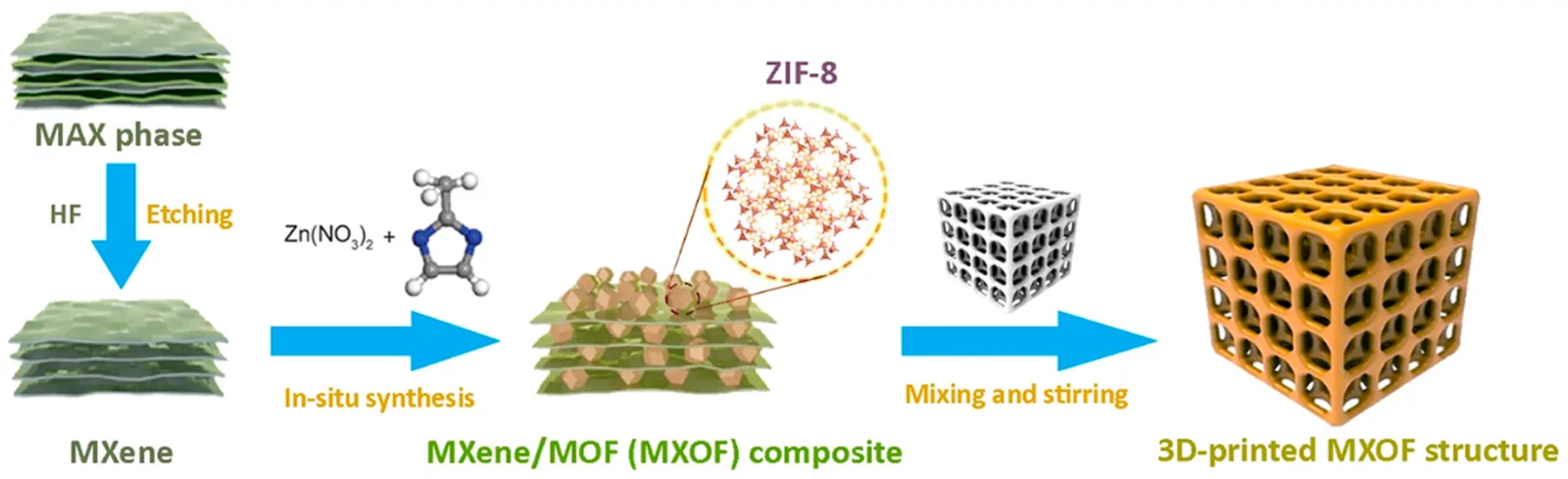
Schematic illustration of the developed method for the fabrication of the 3D-MXOF structure. Reproduced with permission from [85].

In another study, an 80%-porous “vintiles” lattice scaffold was fabricated via DLP, which was post-functionalized by dip-coating in a chitosan-dispersed MXOF composite (ZIF-8 grown in situ on Ti_3_C_2_Tx MXene sheets) to yield the 3D-MXOF adsorbent, as seen in Figure 6 [85]. The purpose was to simultaneously remove two anionic dyes—Direct Red 31 (DR31) and methyl orange (MO)—from a binary aqueous mixture. XRD and FTIR confirmed successful MXene/ZIF-8 hybridization, while BET analysis revealed a striking synergistic surface-area enhancement: the MXOF composite’s specific surface area (1232 m^2^/g) far exceeded that of either the bare MOF (850 m^2^/g) or MXene (11 m^2^/g) alone, which is attributed to ligand–MXene interactions during synthesis. FESEM confirmed uniform, dense coating of MXOF throughout the lattice’s interconnected pores. Adsorption followed pseudo-first-order kinetics, pointing to physisorption as dominant, with a two-stage intraparticle diffusion process, achieving 91.98% removal of DR31 and 84.9% removal of MO. Proposed mechanisms included electrostatic attraction, π–π stacking with the ZIF-8 imidazole rings, Lewis acid interactions at MXene and Zn sites, and physical entrapment within the hierarchical pores. Notably, the monolithic, self-supported format eliminated the need for filtration/centrifugation to recover the adsorbent, and reusability testing over four adsorption–desorption cycles, using ethanol/acetone regeneration, showed minimal efficiency loss (~90% for MO, ~70% for DR31), supporting its potential for industrial textile wastewater applications.

In an interesting case, extrusion-based 3D bioprinting, followed by ionic crosslinking (BaCl_2_) and freeze-drying, was employed to prepare porous aerogels from an alginate/carboxymethylcellulose/gelatin hydrogel pre-loaded with a MOF/MoS_2_ heterojunction [63]. The synthesized composites were then evaluated for both adsorption and photocatalytic degradation of methylene blue (MB) dye. Rheology confirmed shear-thinning, printable behavior, with MOF/MoS_2_ incorporation enhancing mechanical strength and viscosity recovery (up to 77% at 7.5 wt%). Adsorption performance scaled strongly with MOF/MoS_2_ loading–removal efficiency rose from 16.77% (plain hydrogel) to 95.86% (7.5% loading)—with the best-fitting kinetic model shifting from pseudo-first-order (plain hydrogel) to pseudo-second-order as MOF/MoS_2_ content increased. The authors attributed the enhanced uptake to electrostatic interaction with the cationic dye, although a change in kinetic model fit alone cannot distinguish physisorption from chemisorption. Under UV irradiation, all formulations achieved comparable photocatalytic degradation (~81–89%), notably even without added H_2_O_2_, with the unmodified hydrogel performing surprisingly well due to Ba^2+^ ions acting as electron mediators. However, reusability testing over five cycles revealed a trade-off: MOF/MoS_2_-loaded aerogels lost more efficiency (34–42%) and showed structural fracturing compared to the plain hydrogel (23% loss, intact structure), which can be attributed to MOF/MoS_2_ weakening polymer chain interactions and increasing swelling. Table 3 summarizes different post-printing modified adsorbents.

The reusability of post-printing modified adsorbents was also highly variable, and most studies tested only four to six cycles. The highest retention was observed for rigid supports with strongly anchored active phases, such as the chitosan–GO-functionalized SiOC monoliths, which retained more than 92% removal after five NaOH cycles [79], the TiO_2_-functionalized geopolymer lattices, which were stable over five cycles with negligible Ti leaching [75], and the MXOF scaffold, which retained about 90% of its MO removal after four cycles [85]. Moderate retention was found for the MOF/MXene-decorated scaffolds (78–82% after 5–6 cycles) [77] and the silane-grafted chitosan monoliths (about 78% after four cycles) [82], while the GO-decorated PLA filters lost about 32% of their efficiency after the first regeneration before stabilizing [78].

**Figure 7:**
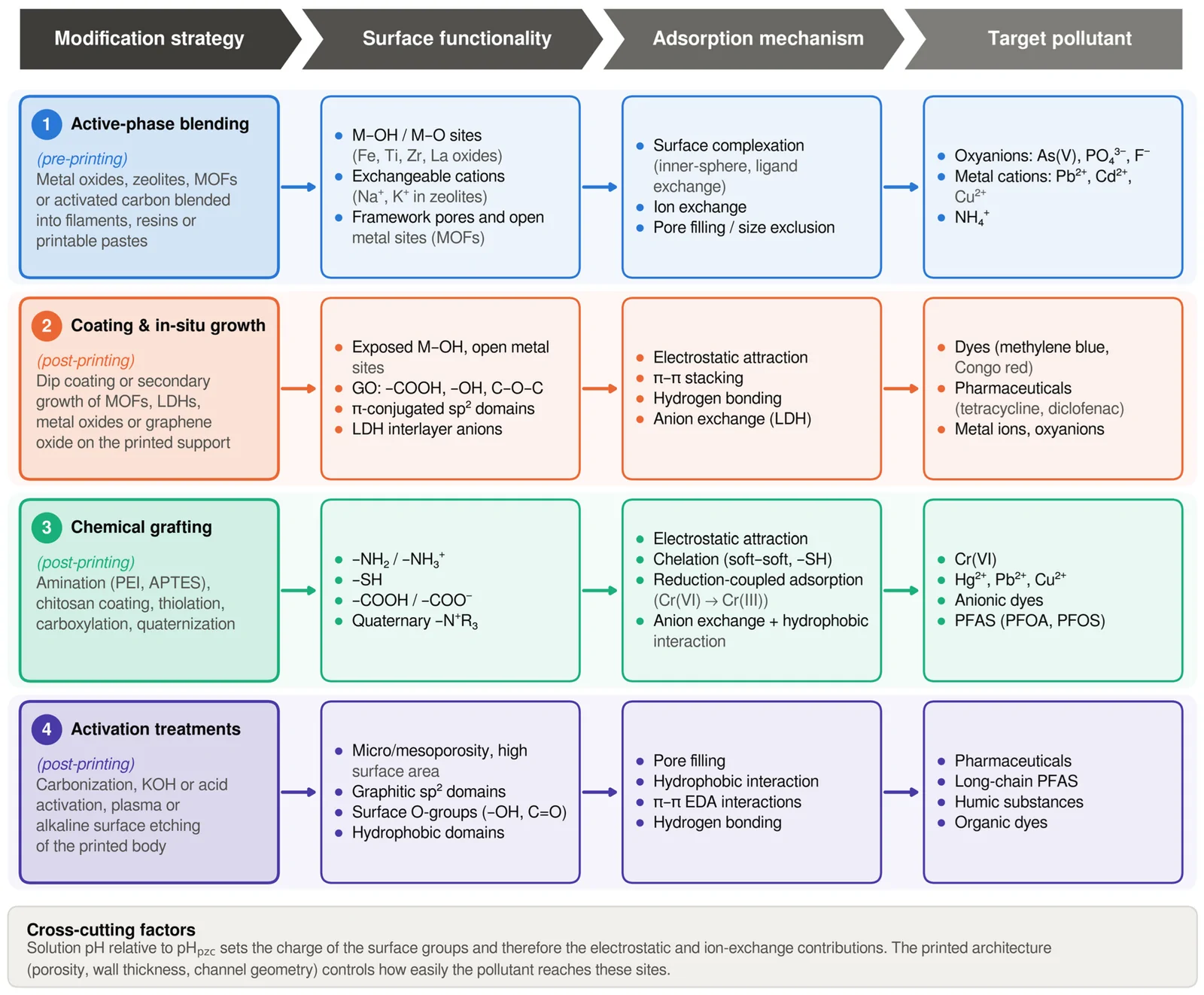
Schematic showing the relationship between the modification strategy, surface functionality, adsorption mechanism, and target pollutant.

Post-printing modified adsorbents were more often evaluated in real matrices. The Cu-MOF/PLA films maintained 90–93% MG removal in tap, lake and river water [76], whereas the GO/AC-DETA scaffolds lost about 20% of their efficiency in real pharmaceutical wastewater [64]. Since the functional groups are directly exposed at the surface, pH strongly governed performance through surface charge and pollutant speciation [78], while coexisting metals could either suppress or enhance uptake, as Cd(II) suppressed and Cu(II) enhanced Pb(II) adsorption on the CS-ZIF-67 scaffolds [84]. Similarly, selectivity was demonstrated by competitive tests for only a few post-printing-modified adsorbents, such as the MOF/MXene-decorated scaffolds, which preferred anionic CR over MB, MO and Sudan III, and Hg(II) over Cu(II) and Cd(II), and the GO/PLA filters, which, when screened against several metal ions, retained mainly cationic Pb(II) and Cd(II) but not the anionic Cr and As species [78]. In most other cases, selectivity was attributed to the surface charge, functional groups or pore size without competitive experiments, as in the case of the size-dependent uptake of MO and DR31 by the MXOF scaffold [85]. Three post-printing modified adsorbents were also tested under continuous flow. The GO/PLA-packed column showed a breakthrough time of 130 h compared with 0.076 h for neat PLA, and its Thomas-model capacity (1.24 mg/g) was close to the batch value, indicating that the batch data could be transferred to flow conditions [78]. In contrast, the CS-ZIF-67 scaffolds removed only 67% of Pb(II) initially and were exhausted after 250 mL [84], while the CNT/TiO_2_-coated geopolymer lattices were evaluated for ciprofloxacin degradation under continuous flow [75]. As pressure drop and bed utilization were not reported, it remains unclear whether the open printed architecture actually improved flow-through operation. Figure 7 presents a schematic representation showing the relationship between the modification strategy, surface functionality, adsorption mechanism, and target pollutant for each of the reported 3D printing methods and modifications.

### 4.6. Comparative Assessment of Pre- and Post-Printing Modification

The studies reviewed in Section 3 and Section 4 show that the choice between pre- and post-printing modification essentially determines where the active phase resides in the printed object, i.e., is distributed throughout the bulk or concentrated at the surface. This single decision affects active-site accessibility, adsorption kinetics, mechanical integrity, durability, and processing requirements. Neither route is universally superior; rather, each offers a distinct set of advantages and limitations, which are compared below.

Pre-printing modification integrates the active phase into the feedstock, so a single printing step yields a functional material with active sites distributed throughout its volume. Because the active phase is embedded in the matrix, it is mechanically anchored and less prone to detachment. For example, no PAC leaching was observed from printed PVDF/PAC filters even after regeneration [49], and hazardous elements in red-mud geopolymer lattices remained immobilized over 28 days [48]. Embedded fillers also proved stable during repeated use, with geopolymer lattices and SLA-printed photocatalytic structures retaining most of their performance over ten cycles [51]. The main limitations stem from the printing process itself. For instance, filler loading is capped by the extrudability and diameter tolerance of filaments in FDM, by ink rheology in DIW, and by the light absorption and scattering of fillers in SLA/DLP, while nozzle temperatures of approximately 180–260 °C in FDM exclude thermally sensitive active phases. Above a certain loading, mechanical properties deteriorate, as shown by the reduced strength of PLA/PBAT scaffolds at high microalgae content [46] and the decrease in tensile strength of TiO_2_-doped resin from 19.4 to 9.9 MPa [52]. Moreover, part of the active phase can be buried within the matrix and remains inaccessible to the pollutant, which in some cases reduced the As(III) capacity of embedded TiO_2_ relative to the free nanoparticles, and about 24 h was required to reach >96% removal [52].

Post-printing modification, in contrast, decouples the fabrication of the structural scaffold from its functionalization, allowing each to be optimized independently. As the active phase is placed directly on the exposed surface, it is readily accessible, and the resulting adsorbents generally reach equilibrium faster; for instance, a PVDF-derived carbon coating on a ceramic scaffold removed BPA completely within 2 h [53]. This route also enables the use of active phases that would not survive the printing conditions or would compromise printability, such as MOFs, MOF/MXene composites, and CNTs grown by CVD and coated by ALD [77], and it has delivered some of the highest capacities in this review, such as 480.95 mg/g for Hg(II) [77]. In certain cases, the spent active layer can be removed and the substrate re-functionalized, as with the ceramic support that was reused after burning off the spent carbon layer [53]. The limitations of post-printing modification relate mainly to the interface between the active layer and the substrate. Coatings and nanoparticles held by weak interactions may delaminate or leach under flow, abrasion, and regeneration, and the surface activation needed to improve adhesion can itself weaken the scaffold, as observed for acetone-treated PLA [80]. Because only the surface is functionalized, the active phase often represents a small fraction of the total mass: the GO/PLA filter showed a capacity of only 1.26 mg/g per filter mass but 58.23 mg/g when normalized to its GO content (~2.16 wt%) [78], and chitosan-grafted monoliths with low surface area reached only approximately 12–13 mg/g [81]. In addition, uniform coating of deep or narrow internal pores is difficult, and additional processing steps, particularly vacuum-based techniques such as CVD and ALD, increase cost and complexity.

The modification route also influences which adsorption mechanisms operate and how effectively they are exploited, since it determines both the functional groups present and their accessibility. Across both strategies, electrostatic interactions were the most frequently reported mechanism and depend on the surface charge of the adsorbent relative to the pollutant: protonated amines of post-grafted chitosan favored anionic methyl orange at acidic pH (88% removal at pH 3 vs. 40% at pH 7) [79], whereas negatively charged GO/PLA filters removed cationic Pb(II) and Cd(II) but not anionic Cr and As species [78]. In pre-printing modification, the mechanisms rely on functional groups of the embedded filler that survive processing, as in microalgae-loaded PLA/PBAT scaffolds, where FTIR showed that the functional groups of the biomass remained intact after extrusion and bound MB through electrostatic interactions and hydrogen bonding [46], and in nanofiber/PLA filters, where carboxyl and amine groups enabled chelation of Cu(II) (up to 234 mg/g) [47]. Post-printing modification, by placing the active phase at the surface, gives direct access to specific binding sites, such as N/S sites coordinating Hg(II) (480.95 mg/g) [77] and ZIF-67 frameworks combining surface complexation of Pb(II) with ion exchange with Co(II) [85], and to aromatic domains enabling π–π interactions, as supported by FTIR band shifts for diclofenac on GO-filled, DETA-grafted scaffolds [64] and proposed for ZIF-8 imidazole rings in MXOF coatings [86]. Pore filling and physical entrapment are expected to contribute in both cases when high-surface-area phases are present, such as PAC in phase-inverted PVDF filters [49] and PVDF-derived carbon or MXOF coatings [53], although direct evidence, such as pore-volume changes after adsorption, was rarely provided. Thus, the two routes rely on the same set of mechanisms, but the selection of the modifier according to the speciation of the target pollutant (e.g., amine-rich groups for anions, oxygen-rich carbons for cations, and N/S-donor phases for metals such as Hg(II)) is as important as the choice between pre- and post-printing modification.

Overall, pre-printing modification is preferable when long-term durability, low leaching, and simple single-step fabrication are the priorities and the active phase is compatible with the thermal, rheological, or optical requirements of the printing technique. Post-printing modification is preferable when the active phase is thermally or chemically sensitive, when fast kinetics are required, or when an expensive active phase should be concentrated where it is accessible. Since the two routes are complementary, hybrid approaches that combine bulk loading with surface functionalization are increasingly used to offset the drawbacks of each, as illustrated by PLA/TiO_2_ units further coated with Ag-loaded TiO_2_ [45] and GO-filled resin scaffolds subsequently grafted with DETA [64]. Finally, direct comparison of the two routes is hindered by the way in which capacities are reported, since values expressed per mass of the whole printed object and per mass of active phase can differ by more than an order of magnitude [78]; reporting both values would allow a fairer assessment of the two strategies.

## 5. Challenges, Research Gaps and Future Directions

The scaling of modified adsorbent 3D printing technology from laboratory level to actual use faces a number of interconnected problems. Uniform loading of functionalized nanomaterials in printed structures becomes more difficult, in addition to the slow speed of direct ink writing, which increases in proportion to the structure size, requiring a compromise between resolution and throughput [88]. Increasing the filler content also raises the viscosity of filaments and inks and makes nozzle clogging more likely, especially with fine nozzles or agglomerated particles. For this reason, a formulation that prints well at laboratory scale cannot simply be transferred to larger structures, where non-uniform drying, sintering shrinkage and cracking become more pronounced. The printer resolution itself is another limiting factor since the regular fused deposition modeling is incapable of creating structures with submicron pores typical for membranes while more advanced technologies such as two-photon polymerization are prohibitively expensive in large-scale production [89]. Post-processing adds its own complications: the heat treatments necessary for sintering of ceramic supports may oxidize pre-loaded magnetic and catalytic nanoparticles; the thorough penetration of nanoparticles into porous structures remains an unsolved problem [90]. In addition, there are issues with the long-term reliability of printed adsorbents as they are exposed to chemical degradation from fouling by oil, biofilm and organic particles that clog active centers; mechanical weakening from wear after each cycle of use; and expansion and weakening of photopolymers in prolonged contact with water. Layer-by-layer printing creates inconsistencies in the microstructure at interfaces and results in additional variability because of differences between the initial CAD model and the printed object. Most importantly, there is very little information on the long-term environmental and regulatory implications of the technology: leaching of nanoparticles under prolonged use is not well studied; there is no regulation of nanomaterial residues in treated water; and life-cycle and ecotoxicity studies that would allow for safe recycling or the disposal of printed filters have yet to be performed. The performance of printed structures has been measured only under laboratory conditions using artificial solutions without consideration of the chemical complexity of actual wastewater.

Regarding practical implementation, only a few of the reviewed studies tested the adsorbents under continuous flow, even shortly and at small scale. Thus, pilot-scale studies with long operation times, larger flow rates and real effluents, together with data on clogging, pressure-drop evolution and cost per volume treated, are still needed before 3D-printed adsorbents can be implemented in practice.

Considering the reviewed studies, a common trend, although mostly qualitative, is established between the print parameters and the adsorption kinetics. First, the position of the active phase that is set by the decision to choose pre- or post-printing modification makes the biggest impact on the speed of the process. The materials in which the active phase is present on the surface of the printed film have reached equilibrium in a matter of minutes to a few hours; for example, more than 90% removal of malachite green was achieved in less than 10 min for Cu-MOF-coated PLA films, while BPA was completely removed within 2 h for the carbon material obtained from PVDF that is coating the ceramic scaffold [53,76]. When the active phase was located inside the solid printed matrix, a longer contact time was needed; e.g., it took 24 h for more than 96% As(III) removal for TiO_2_-doped FCCSs and 24–28 h for PLA lattices due to slow diffusion of the pollutant inside the polymer bulk [52,83]. Similar results were obtained for surface roughening by electron-beam irradiation, which decreased the equilibrium time for chitosan-grafted monoliths from 2 h to 80 min. Another important factor is the geometry of the scaffolds, which can influence the total adsorption performance depending on the available surface area, as seen in the work of Wu et al., in which the removal of CR was between 36.93% for the triangular structure with the lowest specific surface area and 99.13% for the skew-square structure with the largest specific surface area [77]. This set of experiments thus suggests that external film diffusion and diffusion through the printed structure may limit the overall rate. However, a quantitative relation cannot be established, since printing parameters were varied in only a few cases. In addition, the actual printed geometry could be significantly different from the design (up to 42% pore shrinkage according to Fijoł et al. [47]) and the kinetic constants were obtained under various conditions (different concentrations of pollutant and agitation). Thus, future investigations of the adsorption performance of 3D-printed materials should include geometric parameters (filament or strut diameter, pore size, porosity, and geometric surface area), along with kinetic parameters, and should use diffusion models that can distinguish between film and intraparticle resistances. According to Almeida et al., finer nozzles and 45° filaments were associated with a higher surface area and porosity, and hence faster removal of Pb(II) (43% in 15 min) than 90° filaments; however, both geometries eventually removed more than 95% Pb(II) within 2 h [50].

An important question is whether 3D printing really improves performance compared with non-printed adsorbents. The studies reviewed here suggest that 3D printing rarely increases the intrinsic capacity of the active phase. By contrast, printing changes how easily that capacity is accessed and exploited, so a higher removal by a printed adsorbent usually reflects better accessibility or hydrodynamics rather than a higher intrinsic affinity of the active phase for the pollutant. When the active phase is embedded in the printed matrix or diluted in a structural polymer, part of the active sites becomes buried or blocked. As a result, the capacities calculated per total mass of the printed object are usually lower than those of the free powder. This is true even when capacities per mass of active phase remain about the same. The picture is different when the active phase sits on the surface of an open, interconnected structure, as in most post-printing modifications. In this case, site accessibility is maintained and can even improve. This happens because the printed support stops the particles from agglomerating and produces short, well-defined diffusion paths. Still, the clearest advantages of printing are hydrodynamic and practical. Printed monoliths combine a low pressure drop with even flow distribution, and produce sharper breakthrough fronts than packed beds. They also remove the need for filtration or centrifugation to recover the powder, and less active phase is lost during operation and regeneration. However, few studies compare a printed adsorbent with the same active phase in powder form under identical conditions. Such comparisons should become standard practice, with capacities reported both per mass of active phase and per mass of device.

Mass-transfer limitations and mechanical stability also determine whether 3D-printed adsorbents can operate over long periods. Although open channels lower the pressure drop, the dense printed walls slow the diffusion of pollutants to the inner active sites, and breakthrough curves of printed monoliths often show internal mass-transfer limitations [91]. Design can mitigate this: printed geopolymer monoliths showed a shorter mass-transfer zone than beads of identical composition [92], while spiral or chevron channels improved mass transfer and delayed breakthrough at high flow rates, at the cost of a higher pressure drop [93]. Regarding mechanical stability, polymer structures are vulnerable in water, as plasticization and, for PLA, hydrolysis reduce their strength over time, especially at elevated temperatures and in porous parts [94]. Inorganic structures are more durable; printed geopolymer monoliths retained their integrity and performance over eight cycles, whereas beads lasted only three [92]. Stronger anchoring of the active phase, such as covalent crosslinking, can also improve strength and cycling stability [95]. Since most studies report mechanical properties only for as-printed materials and test adsorption in short batch experiments, long-term continuous-flow tests and post-cycling mechanical characterization are needed.

Beyond material issues, there are differences in terms of energy consumption, material efficiency, post-processing requirements, and techno-economic viability, which are, however, not quantified in most of the literature reviewed above. FDM is by far the most economically viable printing technology. Thermoplastics are cheap materials and there is little waste during processing. Composite filaments require compounding and extrusion processes, and the temperature limits the type of thermally sensitive fillers used in the composition as well as their amounts [96]. In DIW, ink is applied at room temperature; this advantage may be deceptive, however. In geopolymer and ceramic inks, energy is consumed in curing, drying or high-temperature sintering after the process, while in hydrogel inks, it is consumed in crosslinking and freeze-drying. SLA/DLP provides the best resolution. It requires expensive photocurable resins and solvent washing in order to remove uncured resin and additional UV exposure [97]. SLS and binder jetting do not require support structures, some of the powders are reusable. Nevertheless, SLS requires a high-powered laser and pre-heating of the powder bed, the powder is deteriorated each time, and binder jetted parts require further sintering or infiltration. There is also the cost and complication of post-printing modification [98]. Post-printing modification includes techniques such as dip-coating and repetitive soaking procedures, but others, such as CVD and ALD, require a vacuum and substantial energy. Organic solvents or reagents are often used, and the amount of solvent consumed is usually not reported. Adsorption capacities do not provide any of this since they are normalized with respect to the mass of the adsorbent. For future research, energy consumption and the input materials should be included; moreover, the yield of material and time required for the treatment of one printed product should be reported. Performance should also be given as cost per unit mass of the removed pollutant or cost per volume of the purified water.

Another issue that should also be emphasized is that the evidence for selective adsorption by 3D-printed adsorbents remains limited. Approximately half of the studies summarized in Table 2 and Table 3 were evaluated only in single-pollutant solutions, where high removal efficiency or capacity reflects affinity for the target pollutant but provides no information on selectivity. Therefore, future studies should proceed in this direction.

Furthermore, of the 36 studies summarized in Table 2 and Table 3, 14 were evaluated only in single-solute synthetic solutions, where a high removal efficiency or capacity reflects affinity for the target pollutant but provides no information on selectivity. Another 12 included multi-solute or competitive tests in synthetic solutions; only one used simulated wastewater; and nine were tested in real waters, of which only five used genuine wastewater (pharmaceutical, textile, mining, e-waste leachate and sewage effluent); while the other four used tap, natural or spiked waters. In these cases, competing ions clearly lowered performance, as with the removal of As(III) from groundwater (33–53%) [52] and of Sr(II) from mining wastewater (22.7%) [67], whereas the effect of natural organic matter, which can compete for active sites and block pores, was rarely examined. Therefore, testing should progress from single-solute solutions to multi-solute solutions, then to simulated and finally to real wastewater, with ionic strength, competing ions and organic matter content reported in each case.

The future directions outlined below differ considerably in maturity, and hybrid approaches that have already been demonstrated for water treatment should be distinguished from concepts that still lack experimental validation. The use of 3D printing in combination with other methods such as electrospinning, atomic layer deposition, vapor-phase deposition, and track etching can provide a means for combining the benefits of freeform structures obtained through additive manufacturing and precise surface modification achieved using one of the aforementioned methods [99]. For example, atomic layer deposition (ALD) is the most mature method for this application: the conformal TiO_2_ coating of CNT-modified 3D-printed geopolymer discussed in Section 4 has already been tested on pharmaceutical mixtures, although only at laboratory scale. In contrast, the combination of printed scaffolds with electrospun nanofibers is well established for tissue engineering [100,101], but its use in adsorption-based water treatment remains largely unexplored, while vapor-phase deposition and track etching of printed adsorbents remain conceptual. Machine learning is at an even earlier stage. One possible approach is the application of machine learning techniques, including computational methods for optimizing pore geometries, choice of functional groups, and lattice design based on contamination removal performance and fluid dynamics inside pores in order to achieve optimal flow rate and contaminant filtration [102]. Although such models are increasingly used to predict the performance of conventional adsorbents, their use in the design of printed adsorbents, with predictions validated by printing and testing, has not yet been demonstrated and should be regarded as an emerging concept. Similarly, the development of stimuli-responsive 4D-printed filters capable of changing their porosity, shapes, or wetting properties depending on pH, temperature, or magnetic field remains speculative for wastewater treatment: stimuli-responsive printed devices have been reported mainly for actuation and analytical applications, such as automated solid-phase extraction, and, to our knowledge, none has yet been evaluated as an adsorbent under realistic wastewater conditions. Progress is further limited by expensive printers and the limited number of stimuli-responsive materials available [103].

Computational design, multimaterial printing, hierarchical architectures and machine learning are often listed as future opportunities for 3D-printed adsorbents. However, they are rarely translated into specific, testable research directions. First, most printed adsorbents are still designed by trial and error, and the effect of geometry on liquid-phase performance has been tested in only a few studies [104]. Second, real effluents usually contain mixtures of cations, oxyanions and organic pollutants, yet most of the printed adsorbents contain a single active phase. Third, the roles of channel geometry and strut porosity are usually not separated, even though both can be tuned independently. A systematic factorial study is needed to define when diffusion inside the walls becomes the limiting step. Finally, machine learning has been applied separately to predict adsorption capacity [105] and ink printability [106], but never to both at once.

Life-cycle assessment (LCA) is the appropriate tool to verify whether the structural and operational advantages of 3D-printed adsorbents translate into environmental benefits, yet no LCA specific to printed adsorbents has been reported in the studies reviewed here. Evidence from other sectors indicates where the burdens are likely to lie. A systematic review of 52 LCA studies on additive manufacturing found that the printing process accounts for almost 80% of the total global warming potential (GWP) of printed products, whereas material-related loads dominate in conventional manufacturing [107]. Similarly, printing electricity accounted for around 70% of the life-cycle GWP of an FDM-printed PLA product, and the impact was closely linked to the printing material employed [108]. For extruded earth- and geopolymer-based materials, which are chemically related to the geopolymer and ceramic inks used in DIW adsorbents, 3D-printed geopolymer made from construction and demolition waste had a lower GWP (488 kg CO_2_ eq) than Portland-cement-based printing (595.6 kg CO_2_ eq) and conventional masonry (533.7 kg CO_2_ eq), but the electricity required to process the waste feedstock represented more than 61% of its GWP [109]. Likewise, the main burden of printed cob arose from the electricity used for printing, with renewable energy and material innovation identified as the main levers for improvement [110]. By analogy, the environmental profile of printed adsorbents is likely to be dominated by printing time and energy and by energy-intensive post-processing (sintering, freeze-drying, CVD/ALD), rather than by the feedstock alone. Future LCAs should therefore follow ISO 14040/14044 [111,112], use a functional unit tied to treatment performance (e.g., mass of pollutant removed or volume of water treated) rather than the mass of adsorbent, and include regeneration cycles and the end-of-life of the spent, pollutant-loaded adsorbent. Environmental safety and regulatory compliance must also be addressed before practical use. The release of nanofillers (e.g., GO, CNTs, MOFs, and metal oxides), the leaching of metal ions from frameworks, residual monomers or photoinitiators from SLA/DLP resins, and micro- and nanoplastics from abraded polymer scaffolds should be quantified under realistic flow and regeneration conditions, together with ecotoxicity tests on the treated water.

Another key aspect is the leaching risk. This depends on the modification route: nanoparticles and coatings added after printing are held by weaker interactions and detach more readily under flow, abrasion, and regeneration than fillers embedded during printing. Released metal ions (e.g., from MOFs), nanocarbons such as GO and CNTs, and photoactive oxides such as TiO_2_ can be toxic to aquatic organisms or carry coexisting pollutants, while spent adsorbents and regeneration eluents form a concentrated hazardous waste stream. The few studies that are presented in this review and quantified leaching reported encouraging results (e.g., immobilized Cr, Cu, and Pb in red-mud geopolymer lattices and Ti release below 0.4 mg/L from TiO_2_-coated CNT/geopolymer over 28 days), but such tests should become standard practice and should be compared against drinking-water or discharge limits. The regeneration method also matters here: thermal regeneration at 400–500 °C, used for the geopolymer lattices, avoids chemical eluents but is energy-intensive, whereas chemical regeneration with HCl, HNO_3_, NaOH or organic solvents requires little energy but produces acidic, alkaline or solvent-rich eluents that need further treatment, and neither the energy demand nor the volume of eluent was usually reported.

Finally, in order to convert these laboratory discoveries into actual technology, it will require continuous interaction among researchers, industry, and water management agencies to evaluate their effectiveness in real-world applications over time. This requires going beyond simple experiments with one pollutant under one set of conditions to standardize testing methods, such as measuring model fit using χ^2^, RMSE, and R^2^. Equally importantly, a good fit to the kinetic or isotherm models does not by itself establish a molecular adsorption mechanism. The PSO model in particular fits a wide range of datasets irrespective of the underlying process, and its frequent equation with “chemisorption” is not justified on kinetic grounds alone, while Langmuir and Freundlich parameters describe equilibrium behavior rather than the nature of the binding sites. Mechanistic claims should therefore be supported by direct evidence, such as FTIR or XPS shifts of the functional groups involved before and after adsorption, surface-charge data (zeta potential or pHpzc) interpreted against pollutant speciation, and competitive-ion or ionic-strength experiments, as was carried out in several of the studies discussed in Section 4. In order to scale up the fabrication process faster, it is possible that methods such as binder jetting may become necessary, in addition to quality control systems for consistent batches. Due to the sensitivity of nanomaterials in drinking water, it is important to report their leaching behaviors as well as the new regulations regarding nanomaterials.

## 6. Conclusions

Overall, this review focused on the synthesis and modification of 3D-printed absorbents, categorized based on whether modification took place before the printing process or after. For neither approach was there a clear advantage, because while pre-printing modification typically allowed for a higher loading of the active phase (since the active phase is uniformly distributed within the volume of the 3D-printed object, whereas post-printing modification only modifies a surface layer), this was at the expense of printability and the strength of the structure, with adsorption capacity and mechanical properties being compromised when the loading of the filler exceeded a certain threshold. On the other hand, post-printing modification generally involved lower active-phase loading but offered more accessible active sites and faster kinetics, while maintaining the structural stability of the scaffold and, in some cases, even enabling the decoupling of the structural material from the active layer—the coatings were able to be removed, and the scaffold was able to be re-functionalized.

With these complementary capabilities, the way forward will not lie in favoring one strategy over the other but rather in merging the two; some of the top adsorbents reported in this review utilized a combination of pre-print loading of the functional ink and post-print functionalization (for instance through surface grafting, crosslinker exchange, or secondary coating) to benefit from the volumetric capacity of pre-printing, in addition to the surface control and recyclability offered by post-printing techniques.

Beyond this general comparison, several critical findings emerged from the reviewed studies. The main advantages of 3D-printed adsorbents were associated with their hydrodynamic and operational behavior, namely, the low pressure drop, the sharper breakthrough fronts and the elimination of post-treatment separation of the adsorbent. Secondly, reusability was governed primarily by the nature of the support, with ceramic and geopolymer structures retaining most of their performance after approximately ten cycles, whereas polymer-based structures, and particularly coatings deposited on PLA or on mechanically weak aerogels, exhibited a more pronounced loss of capacity. Finally, the limited studies conducted in real water matrices demonstrated that pH, competing ions and dissolved organic matter can significantly reduce removal efficiency.

In light of these findings, future research should be directed towards specific priorities. Printed adsorbents should be systematically benchmarked against the same active phase in powder form under identical experimental conditions, with adsorption capacities reported both per mass of active phase and per mass of device. Moreover, the actual printed geometry (strut diameter, pore size and porosity) should be characterized and reported alongside kinetic data, in order to quantify the effect of structural design on mass transfer. Finally, evaluation in real effluents under continuous-flow conditions, even at pilot scale, along with treatment cost and life-cycle impact, is essential.

## Figures and Tables

**Table 1:** Overview of the main 3D-printing technique.

Technique	Feedstock Materials	Resolution	Advantages	Limitations	Wastewater Treatment	Ref.
Fused Deposition Modeling (FDM/FFF)	PLA, ABS, PETG, TPU	100–300 µm	Low cost, wide material range, easy post-loading of fillers	Layer anisotropy, limited resolution, thermal degradation of additives at nozzle temperatures (~180–260 °C), low intrinsic porosity of printed structures	Most widely used for low-cost porous adsorbent scaffolds; easy to blend adsorptive fillers into filament; low-cost porous filters and scaffolds for dyes (MB, MG) and heavy metals (Cu(II), Cd(II)); pre-printing composite filaments (biomass, nanocellulose, GO) and post-printing coating, solvent activation, or in situ MOF growth	[22]
Direct Ink Writing (DIW)	Ceramic pastes, hydrogels, biopolymers	100 µm–1 mm	Compatible with functional/composite inks at room temperature, good for hierarchical porosity	Requires careful rheology control, slow build speed	Well-suited for directly printing functional adsorbents (e.g., MOF- or clay-loaded structures) without high-temperature processing; monoliths and aerogels for heavy metals and radionuclides (Cu(II), Pb(II), U(VI), Au(III)), dyes (MB), and pharmaceuticals (ibuprofen, ciprofloxacin); predominantly pre-printing functional inks (alginate/GO, MOFs, biochar, geopolymers), with post-printing ALD or nanoparticle decoration	[23]
Stereolithography (SLA)/Digital Light Processing (DLP)	Acrylate/epoxy resins, ceramic-filled resins	25–100 µm	High resolution, smooth surface finish, complex geometries	Limited material chemistry, resin often needs post-curing, potential cytotoxic residues	High-surface-area lattices and ceramic monoliths for As(III), Pb(II), Hg(II), dyes (MO, CR), and organics (BPA, diclofenac); pre-printing resin doping (TiO_2_, nanochitosan, GO) and post-printing dip-coating, grafting, or MOF/PDA deposition	[24]
Selective Laser Sintering (SLS)	Nylon (PA11/12), TPU	80–150 µm	No support structures needed, good mechanical strength	High equipment cost and energy demand (laser, pre-heated powder bed), rough surface finish, limited powder reusability	Less common for adsorbents but useful for robust, reusable filter housings and supports	[25]
Binder Jetting (BJ)	Ceramics, metals, sand, ceramic-metal composites	50–200 µm	Room-temperature processing, wide material compatibility, can print porous ceramics directly	Green parts require post-processing (sintering/infiltration) for strength	Attractive for producing porous ceramic adsorbents with tunable porosity via binder/powder ratio	[26]

**Table 3:** Summary of post-printing modifications on adsorbent materials.

Name	3D Printing Technology	Type of Modification	Pollutant	Adsorption Conditions	Qm (mg/g)	Regeneration	Selectivity	Ref.
3D-CS-ZIF-67 filter	SLA	Dip-coating of printed scaffold in chitosan/ZIF-67 composite solution	Pb(II)	Dosage: n.r. pH: 6 Initial concentration: 50 mg/L contact time: 360 min	5.47	Not explicitly tested (fixed-bed exhaustion at 250 mL)	Cu(II) enhanced Pb uptake; Cd(II) suppressed it (competing-ion study)	[84]
3D-MXOF structure	DLP	Dip-coating of printed lattice in chitosan-dispersed MXene/ZIF-8 (MXOF) composite	Direct Red 31 (DR31), Methyl Orange (MO)	Dosage: 48 g/L pH: 4 Initial concentration: 30 mg/L contact time: 60 min	1.27–1.28 (DR31); 1.38–1.4 (MO)	4 cycles; ~90% (MO)/~70% (DR31) retained	Size-selective: MO (smaller) adsorbed faster/more than DR31	[85]
TiO_2_(KH-570)/PLA water-treatment unit (Ag-TiO_2_ coating)	FDM	UV-curable Ag-loaded TiO_2_ photoresin coating applied to the printed surface	Methylene blue (photocatalytic); *E. coli*	Dosage: n.r pH: n.r. Initial concentration: 10 mg/L contact time:	n.r.	Not tested	Not tested	[45]
PVDF-derived carbon (PVDF/C)-coated ceramic device	SLA (ceramic resin)	PVDF dip-coating + N_2_ calcination (carbonization) on the sintered ceramic scaffold	Bisphenol A (+phenols, pharmaceuticals, dyes)	Dosage: 0.58 g/L pH: 6 Initial 20 mg/L contact time: 1440 min	122.5	10 cycles; >90% retained; support itself is re-coatable after decoking	Competitive test, non-selective (broad-spectrum): ~100% individual and simultaneous removal of 6 additional pollutants	[53]
3D-printed geopolymer + CNTs/TiO_2_ (ALD)	DIW	Catalyst-free CNT growth (thermal CVD, using red mud’s Fe) + ALD TiO_2_ shell on the printed lattice	Ciprofloxacin (+sulfamethoxazole, acetaminophen, ketoprofen mixture)	Dosage: n.r. pH: 6.5 Initial concentration: 10 mg/L contact time: 60 min	n.r.	5 cycles; 91.8 → 87.4% (partial recovery with ethanol wash)	Competitive test (photocatalytic, four-drug mixture): 79–93% removal; pH dependence linked to CIP speciation	[75]
Cu-MOFs/PLA films	FDM	Stepwise in-situ growth of Cu-MOF crystals (H_3_BTC/TEA + Cu(NO_3_)_2_ soaks, 1–8 cycles)	Malachite green (MG)	Dosage: n.r. pH: n.r. Initial concentration: 10 mg/L contact time: 10 min	n.r.	5 cycles (acetone wash), >60% retained; PLA scaffold itself regenerable (HCl strip + re-coat)	Not tested	[76]
Pd-nanoparticle-decorated TiO_2_ scaffold	DIW	Hydrothermal growth of Pd NPs on the silane-activated, annealed TiO_2_ scaffold	4-nitrophenol (catalytic reduction, not adsorption)	Dosage: n.r. pH: n.r. Initial concentration: n.r. contact time: n.r.	Not applicable	20 cycles; no obvious deactivation, ~0.04 wt% Pd leaching	Not tested	[78]
PDA/MOFs(ZIF-67/ZIF-8)/MXene/3DS	SLA	Dip-coating in MOFs/MXene composite, fixed onto the printed scaffold with polydopamine (PDA)	Congo Red (CR), Hg(II)	Dosage: n.r. pH: 7(Cr), 6(Hg(II)) Initial concentration: 20mg/L (CR), 50mg/L (Hg(II)) contact time: 120 min	77.95 (CR); 480.95 (Hg)	5–6 cycles; 78–82% retained	Selective for anionic CR (vs. MB, MO, Sudan III) and Hg(II) (vs. Cu, Cd)	[77]
GO/PLA filter (Cd)	FDM	Acetone-induced surface roughening + GO doping by sonication	Cd(II) (+As, Co, Cr, Cs, Cu, Fe, Hg, Ni, Pb, Zn screened)	Dosage: 8 g/L pH: 6 Initial concentration: 10 mg/L contact time: n.r.	1.26 (filter); 58.23 (per g GO)	5 cycles (HNO_3_); drop after cycle 1, then stable	Cationic-selective: Pb (95.9%) > Cd (86.6%) > others; anionic Cr/As excluded	[78]
Chitosan–GO grafted SiOC ceramic monolith	DLP (LCD-SLA, preceramic polymer)	Silane (GLYMO) grafting of chitosan–GO composite onto the pyrolyzed SiOC ceramic	Methyl Orange (MO)	Dosage: 0.25 g/L pH: 3.0 Initial concentration: 50 mg/L contact time: 80 min	182.25	5 cycles (NaOH); >92% retained	Not tested	[79]
Silane-grafted nanochitosan + electron-beam irradiation	mSLA	Silane-grafted nanochitosan coating + post-print electron-beam irradiation (50–200 cGy)	Methyl Orange (MO)	Dosage: n.r. pH: 6.3 Initial concentration: 50 mg/L contact time: 80 min	12.2	Not cycle-tested (weight-loss stability only, <1%)	Not tested	[81]
Chitosan-grafted PEGDA monolith (RSM/BBD-optimized)	DLP (mSLA)	Silane-grafted chitosan coating; grafting conditions optimized by Box–Behnken design	Methyl Orange (MO); real textile wastewater	Dosage: n.r. pH: 6.3 Initial concentration: 50 mg/L contact time: 120 min	13.21	4 cycles; ~78% retained after cycle 2	Not tested; BOD reduction 62.4%, COD reduction 2%	[82]
BR-GO-DETA/BR-AC-DETA Gyroid composite	SLA (vat photopolymerization)	Post-print DETA amine grafting (nucleophilic addition + amide coupling) on the GO/AC-loaded lattice	Diclofenac	Dosage: 1 g/L pH: 7 Initial concentration: 20 mg/L contact time: 1440 min	91.5 (GO); 12.3 (AC)	5 cycles (GO)/3 cycles (AC); 80–98% retained after cycle 2	Not directly tested; ~20% loss in real pharmaceutical wastewater	[64]
GO/PLA filter (acetone pre-treatment optimization)	FDM	Acetone pre-treatment (95%, undried) to enhance GO doping onto printed PLA	#Methylene blue (MB)	Dosage: 32 g/L pH: 6.5 Initial concentration: 50 mg/L contact time: 1440 min	4.84 (filter); ~200 (per g GO)	Not tested	Not tested	[80]
EDTA-dealuminated 3D-printed metakaolin geopolymer (0.1EDTA8h)	DIW (robocasting)	Post-print acid dealumination (EDTA, 0.01–0.15 M, 8–24 h) of a plain metakaolin-geopolymer lattice	Methylene blue (MB)	Dosage: 5 g/L pH: 10.5 Initial concentration: 110 mg/L contact time: 480 min	30.86	10 cycles (thermal, 500 °C); >93% retained	Not tested	[86]
CelloZIF (TOCNF/ZIF-8, binder-free)	FDM-type extrusion (TOCNF ink)	Post-print soaking of printed TOCNF/Zn^2+^ or TOCNF/Hmim scaffold in the complementary reagent to grow ZIF-8 crystals in situ	CO_2_ (gas capture); Methylene blue (anionic-dye selectivity vs. Rhodamine B); Cu(II), Co(II)	Dosage: 2 g/L pH: n.r. Initial concentration: 50 mg/L (dyes); 1000 mg/L (Cu, Co) contact time: 10 min (dyes); 10 min (metals)	27.7 (CO_2_); 101.2 (Cu^2+^); 328 (Co^2+^)	CO_2_ adsorption recyclable over several cycles; dye/metal reuse not tested	Highly selective for anionic MeB over cationic RhB	[87]

* n.r. = not reported.

## Data Availability

No new data were created or analyzed in this study. Data sharing is not applicable to this article.
